# An annotated checklist of grasshoppers (Orthoptera, Acridoidea) from Mongolia

**DOI:** 10.3897/BDJ.11.e96705

**Published:** 2023-03-13

**Authors:** Enkhtsetseg Gankhuyag, Altanchimeg Dorjsuren, Eun Hwa Choi, Ui Wook Hwang

**Affiliations:** 1 Department of Biology, Teachers College, and Institute for Phylogenomics and Evolution, Kyungpook National University, Daegu 41566, South Korea Department of Biology, Teachers College, and Institute for Phylogenomics and Evolution, Kyungpook National University Daegu 41566 South Korea; 2 Institute of Biology, Mongolian Academy of Sciences, Ulaanbaatar 133330, Mongolia Institute of Biology, Mongolian Academy of Sciences Ulaanbaatar 133330 Mongolia; 3 College of Life Sciences, Inner Mongolia University, Hohhot, 010031, China College of Life Sciences, Inner Mongolia University Hohhot, 010031 China; 4 Institute for Korean Herb-Bio Convergence Promotion, Kyungpook National University, Daegu 41566, South Korea Institute for Korean Herb-Bio Convergence Promotion, Kyungpook National University Daegu 41566 South Korea; 5 Institute of Phylogenomics and Evolution, and Department of Biology, Teachers College Kyungpook National University, Daegu 41566, Republic of Korea Institute of Phylogenomics and Evolution, and Department of Biology, Teachers College Kyungpook National University Daegu 41566 Republic of Korea; 6 School of Industrial Technology Advances, Kyungpook National University, Daegu 41566, South Korea School of Industrial Technology Advances, Kyungpook National University Daegu 41566 South Korea; 7 Phylomics Inc., Daegu 41910, South Korea Phylomics Inc. Daegu 41910 South Korea

**Keywords:** distribution, fauna, natural zone, Pamphagidae, Acrididae, Dericorythidae, Mongolia

## Abstract

**Background:**

Grasshoppers (Acridoidea, Orthoptera) are the dominant herbivores in grassland ecosystems worldwide. They can increase rangeland productivity by stimulating plant growth and accelerating nutrient cycling. This article presents a comprehensive checklist of grasshoppers in Mongolia. Until then, the available information was very scattered, based on old studies of Mongolian grasshoppers, recorded in a few international catalogues and databases, individual records and research work on agroecosystem communities. However, the available information on the composition of the Orthopteran fauna in Mongolia was sometimes unclear or non-existent and these dubious data were excluded from the present study. In addition, the grasshopper distribution analysis used the standardised personal collection of D. Altanchimeg. We also present a list of grasshoppers, as well as their distribution and abundance, in countries adjacent to Mongolia, such as Russia, China and South Korea. The surveys covered six types of natural zones: high mountain, taiga, forest-steppe, steppe, desert steppe and desert; desert steppe and steppe zones are the most widely distributed. We hope to have contributed significantly to the study of the distribution of grasshopper species in all these natural zones.

**New information:**

In this study, a total of three families of Acridoidea belonging to eight subfamilies, 17 tribes, 52 genera and 128 species are reported for the various natural zones. The recorded species belong to eight subfamilies: Gomphocerinae are the most numerous with 56 species recorded, followed by Oedipodinae (51 species), Thrinchinae (nine species), Melanoplinae (six species), Calliptaminae (three species), Dericorythinae, Acridinae, Egnatiinae (one species each).

## Introduction

Locust and grasshoppers (Orthoptera, Acridoidea) are essential herbivores in grassland ecosystems worldwide (Latchininsky et al. 2011, Fang et al. 2015). They aid in plant growth and nutrient cycling and play an important part in food chains (Kietzka et al. 2021). However, locust and grasshopper outbreaks are considered a global problem. They can destroy grasslands and crops and inflict severe economic impacts on crops and rangelands (Hewitt 1977, Gupta 1983, Lockwood and Lockwood 2008, Latchininsky et al. 2011, Zhang et al. 2019, Lecoq and Zhang 2019, Lecoq and Cease 2022). A few grasshopper species have been proposed as ecological indicators of ecosystem health as they are susceptible to changes in land use and climate (Marini et al. 2008, Fartmann et al. 2012, Bazelet and Samways 2012, Uchida and Ushimaru 2014). Climate change and geographical characteristics are critical factors that determine grasshopper population growth. The grasshoppers (Acridoidea) are the largest superfamily of the orthopterans with 28 families, 140 subfamilies, 265 tribes, 2,459 genera (57 subgenera) and 10,531 valid species (1,951 subspecies) distributed throughout the world, except Antarctica (Cigliano et al. 2022). Mongolia is a landlocked country with diverse terrain, surrounded by mountains to the north and west and the forest-steppe of the Gobi Desert to the south. The majority of its land is covered by grassland steppe. Mongolia has six main natural zones and belts (that are divided into sub-divisions): alpine (high mountain) and mountain taiga, mixed and deciduous forests, steppe, desert steppe (Gobi) and desert zones (Yembuu 2021). Mongolia has a high elevation and cold and dry climate (Worden and Savada 1991, Gombobaatar et al. 2014).

Of these species, Mongolia contains 128 species in 52 genera and three families (Pamphagidae, Dericorythidae and Acrididae). In the early 1960s, several foreign and Mongolian researchers started undertaking expeditions for the Mongolian insect species checklist. Some of these expeditions were undertaken collaboratively with other countries, such as Poland (1962–1964), Hungary (1963–1968), Germany (1962–1964), the Czech Republic (1965–1966) and Russia (1967–1987). This research facilitated the study of insect distribution (physical and geographical distribution) in the different provinces of Mongolia and collected several grasshopper specimens. Referring to Rentsendorj and Khodroi (2020), the study on the list of grasshoppers in Mongolia was undertaken from 1951 to 2019 and a list of 110 species was established. Of them, three different families and 48 genera between 1951 and 1989 (Mistshenko and Bey-Bienko 1951, Mistshenko 1968, Günther 1971, Chogsomzhav 1971, Chogsomzhav 1974b, Chogsomzhav 1977, Chogsomzhav 1989, Gorochov et al. 1989) and 81 species of two families and 33 genera between 1990–2019 (Batkhuyag 1995, Batnaran et al. 1999, Batnaran 2008, Ganbold 2009, Altanchimeg et al. 2014, Altanchimeg and Nonnaizab 2013, Batkhuyag et al. 2014, Gandulam 2016, Otgonchimeg 2017) were added to the list. Moreover, 69 short-horned grasshopper species, which belonged to two families and 31 genera, were identified between 2013 and 2019 in Mongolia (Rentsendorj and Khodroi 2020). Recently, the species list of Mongolian grasshoppers was updated to three families, 49 genera and 127 species (Batkhuyag and Batnaran 2021). However, as this species list included uncertain specimen records and unclear sources, the accuracy of these sources needed to be checked.

The present study is the first comprehensive study to update the diversity of Mongolian grasshoppers (Acridoidea), including information on species traits and distribution by natural zones. To create this annotated checklist, we reviewed all published materials related to short-horned grasshopper species that were newly discovered in Mongolia since the 1930s, followed by a taxonomic analysis using important source information from the Orthoptera Species File (Cigliano et al. 2022). The current study's findings will give fundamental information regarding the grasshopper diversity of Mongolia. Furthermore, it can inspire local scientists interested in topics such as ecology, biology, medicine, agriculture and education.

## Materials and methods

The list presented in the present paper is based on literature records of grasshopper species in Mongolia available up to May 2022. The taxa that were reported from Mongolia are listed taxonomically by subfamily and alphabetically within each subfamily, tribe and genus. Each species was examined in Mongolia and the citation of the first or most reliable reference to support this record is provided. First, the references to recorded species in Mongolia reported in the Orthoptera Species File online version 5.0/5.0 (Cigliano et al. 2022), Institute of Biology, Mongolian Academy of Sciences (MAS) (Chogsomzhav 1975, Altanchimeg et al. 2014), Plant Protection Research Institute of Mongolia (Batkhuyag et al. 2014) and the grasshopper list in Mongolia from the *Mongolian Journal of Agricultural Sciences* were checked and, if erroneous, corrected in the current lists. Changes in systematic status and synonymies have been proposed by (Chogsomzhav (1989), Altanchimeg (2011) and Batkhuyag and Batnaran (2021).

The current work is an annotated study of grasshoppers (Acridoidea) in Mongolia, with an emphasis on a rare and unknown grasshopper species. Except for long-horned grasshoppers and crickets, this research systematises Acridoidea. Likewise, the list is used in taxonomic sequences by superfamily, family, subfamily, tribe, genus and species. Depending on the natural zone where the grasshopper was collected, they belong to that area. Grasshopper distribution in six different natural zones, distribution of grasshopper species in neighbouring countries on the northern border with Russia and south with China and comparison with the South Korean population were performed using the Biodiversity Pro 2.0 programme. In addition, in all cases, the similarity matrix of the Bray-Curtis cluster analysis dendrogram was used (single link). The grasshopper distribution in the six different natural zones was determined using ArcGIS ArcMap 10.7.1. The grasshopper's geographical distribution was drawn, based on the Mongolian steppe figure (Mohamed, A and Kimura 2014). The species registered on the European Red List were also divided into categories using IUCN Red List Categories and Criteria (IUCN 2022).

### Institutional Abbreviations

**(B.-Ulg.)** = Bayn-Ulgii

**(Zav.)** = Zavkhan

**(Khuvs.)** = Khuvsgul

**(A.-khang)** = Arkhangai

**(Bulg.)** = Bulgan

**(Orkh.)** = Orkhon

**(Sel.)** = Selenge

**(Da.**) = Darkhan-Uul

**(Khent.)** = Khentii

**(S.-baat.)** = Sukhbaatar

**(Dо**) = Dornod

**(G.-alt.)** = Gobi-Altai

**(B.-khong.)** = Baynkhongor

**(U-khang)** = Uvurkhangai

**(Du.-govi)** = Dundgovi

**(U.-govi**) = Umnugovi

**(Do.-govi)** = Dornogovi.

(*) = Endemic species of Mongolia

(+) = Geographical distribution of the natural zones

(★) = Indicator species of the geographical natural zones

(-) = poor species

## Checklists

### Checklist of Mongolian grasshopper

#### Acrida kozlovi

(Mistshenko, 1951)

E3BF5026-0A59-5F97-85EC-0DD9F8B02D5B

http://orthoptera.speciesfile.org/Common/basic/Taxa.aspx?TaxonNameID=1111388

##### Native status

**Distribution in the natural zone**: Desert steppe and desert.

##### Distribution

**in Mongolia**: U.-govi., Uvs. Mistshenko (1968):489, Chogsomzhav (1989):90, Myagmar et al. (2019):56, Batkhuyag and Batnaran (2021):48.

**Global distribution**: China, Inner Mongolia, Ningxia, Mongolia, Russia (Mistshenko and Bey-Bienko 1951, Chogsomzhav 1989).

#### Arcyptera (Arcyptera) albogeniculata

Ikonnikov, 1911

5914DC73-169C-5F7B-BBBF-1CBA88892328

http://orthoptera.speciesfile.org/Common/basic/Taxa.aspx?TaxonNameID=1105385

##### Native status

**Distribution in the natural zone**: Forest steppe.

##### Distribution

**in Mongolia**: Tuv., S.-baat., Do., Khovd. Ikonnikov (1911):250, Cejchan and Maran (1966):179, Steinmann (1967):108, Mistshenko (1968):490, Chogsomzhav (1970):127, Günther (1971):114, Altanchimeg and Nonnaizab (2013), Batkhuyag and Batnaran (2021):64.

**Global distribution**: China, South Korea, Mongolia (Ikonnikov 1911, Storozhenko and Paik 2007).

#### Arcyptera (Pararcyptera) meridionalis

Ikonnikov, 1911

0B9C68DA-7240-5B3E-93C1-BD429531558E

http://orthoptera.speciesfile.org/Common/basic/Taxa.aspx?TaxonNameID=1105423

Arcyptera flavicosta sibirica Uvarov (1914):170.

##### Native status

**Distribution in the natural zone**: Forest steppe, steppe and desert steppe.

##### Distribution

**in Mongolia**: Uvs, Khuvs., Bulg., Tuv, Khent., S.-baat, B.-khong, U-khang., Du.-govi., U.-govi., Sel., Khent., A.-khang., Do. Uvarov (1914):170, Pylnov (1916):278, Chogsomzhav and Shurovenkov (1963):61-63, Cejchan and Maran (1966):180, Mistshenko (1968):490, Chogsomzhav (1968):56, Chogsomzhav (1970):127, Chogsomzhav (1974b):27, Chogsomzhav (1989):91, Storozhenko and Paik (2007):168, Sergeev et al. (2019):21, Batkhuyag and Batnaran (2021):65.

**Global distribution**: South Korea (Storozhenko et al. 2015), Tuva, S Siberia (from Tuva to Dauria and Yakutia), the southern part of the Russian Far East, Mongolia, NE China, N Korea (Sergeev et al. 2019).

#### Arcyptera (Pararcyptera) microptera

(Fischer von Waldheim, 1833)

48EE2A60-D451-5CD2-BCDB-3630C070A333

http://orthoptera.speciesfile.org/Common/basic/Taxa.aspx?TaxonNameID=1105407

##### Native status

**Distribution in the natural zone**: Forest steppe, steppe and desert steppe.

##### Distribution

**in Mongolia**: Khovd. Chogsomzhav (1972):160, Sergeev (1995):245, Sergeev et al. (2009):108, Altanchimeg (2011):16, Altanchimeg and Nonnaizab (2013), Batnaran et al. (2016):32, Sergeev et al. (2019):21, Batkhuyag and Batnaran (2021):64.

**Global distribution**: S Europe, W Siberia, S Krasnoyarsk Region, Caucasus, Kazakhstan, NW Mongolia, NW China (Sergeev et al. 2019).

#### Arcyptera (Arcyptera) fusca

(Pallas, 1773)

E7FFEF61-0161-59C6-BB64-3D2546379DD9

http://orthoptera.speciesfile.org/common/basic/Taxa.aspx?TaxonNameID=47973

Gryllus cothurnatus Creutzer (1799):129.
Gryllus (Locusta) nympha Stoll (1813):23.
Arcyoptera stollii Fieber (1853):99.
Gryllus (Locusta) variegatus Sulzer (1776):84.
Gryllus versicolor Gmelin (1789):2082.

##### Native status

**Distribution in the natural zone**: Forest steppe.

##### Distribution

**in Mongolia**: Zav., Khuvs., A.-khang., Sel., Tuv., Khovd, U-khang, Du.-govi., B.-khong. Pylnov (1916):278, Cejchan and Maran (1966):179, Steinmann (1967):109, Mistshenko (1968):490, Chogsomzhav (1968):57, Chogsomzhav (1970):127, Chogsomzhav (1989):91, Batkhuyag (1995):29, Childebaev and Storozhenko (2001), Sergeev et al. (2009):108, Altanchimeg and Nonnaizab (2013), Altanchimeg et al. (2013b):65, Sergeev et al. (2019):20, Popova et al. (2020):599, Batkhuyag and Batnaran (2021):64.

**Global distribution**: Tuva, southern part of European Russia, S Siberia up to Sakha (Yakutia), Amur Region, mountains of S Europe, Moldova, Ukraine, Caucasus, Kazakhstan, Mongolia, NE China (Sergeev et al. 2019).

#### Andrea gorochovi

Mistshenko, 1989

B5BA9B02-A05F-5C61-BFEA-18B1B0060FAA

http://orthoptera.speciesfile.org/Common/basic/Taxa.aspx?TaxonNameID=1104197

##### Native status

**Distribution in the natural zone**: Desert steppe.

##### Distribution

**in Mongolia**: B.-Khong Gorochov et al. (1989):99,102, Batkhuyag and Batnaran (2021):103.

#### Chrysochraon dispar

(Germar, 1834)

AE0CC795-9B5A-5F7D-A4C1-B5523CB1A5D5

http://orthoptera.speciesfile.org/Common/basic/Taxa.aspx?TaxonNameID=1106359

##### Native status

**Distribution in the natural zone**: High mountain, taiga, forest-steppe, steppe, desert steppe, and desert.

##### Distribution

**in Mongolia**: Sel. Chogsomzhav (1972):157, Chogsomzhav (1989):93, Altanchimeg and Nonnaizab (2013), Altanchimeg et al. (2013b):65, Batkhuyag and Batnaran (2021):57.

**Global distribution**: Tuva, N Eurasia (except the extreme North), Caucasus, a mountain of Middle Asia, Mongolia (Sergeev et al. 2019).

#### Euthystira brachyptera

(Ocskay, 1826)

3CF10C63-E086-5E94-AC22-BEA597E9E2E9

http://orthoptera.speciesfile.org/common/basic/Taxa.aspx?TaxonNameID=1106449

##### Native status

**Distribution in the natural zone**: High mountain, taiga, forest-steppe, steppe, desert steppe and desert.

##### Distribution

**in Mongolia**: Sel. Chogsomzhav (1969a):77, Chogsomzhav (1972):157, Chogsomzhav (1989):93, Altanchimeg and Nonnaizab (2013), Altanchimeg et al. (2013b):65, Batkhuyag and Batnaran (2021):57.

**Global distribution** Tuva, N Eurasia (the southern part of the forest zone, the forest-steppe and steppe zones), Mongolia (Sergeev et al. 2019).

#### Mongolotettix mistshenkoi

Chogsomzhav, 1974

D24FD5C8-8F6C-5E32-A6CF-4512500207BD

http://orthoptera.speciesfile.org/Common/basic/Taxa.aspx?TaxonNameID=1107086

##### Native status

**Distribution in the natural zone**: Steppe and desert steppe.

##### Distribution

**in Mongolia**. Do.-govi. Chogsomzhav (1974a), Chogsomzhav (1975):42, Altanchimeg (2011):16, Batkhuyag and Batnaran (2021):58.

#### Mongolotettix japonicus

(Bolívar, 1898)

9C1B287B-6DDF-5D49-8A2C-C5AE85F7C30A

http://orthoptera.speciesfile.org/Common/basic/Taxa.aspx?TaxonNameID=1107087

##### Native status

**Distribution in the natural zone**: Taiga, forest-steppe, steppe and desert steppe.

##### Distribution

**in Mongolia**: Uvs, Khuvs., A.-khang., Bulg., Sel., Tuv., Khent., S.-baat., Do., U-khang., U.-govi. Chogsomzhav (1989):93, Batkhuyag and Batnaran (2021):58.

**Global distribution**: South Korea, Mongolia (Storozhenko and Paik 2007), Japan (Bolívar 1898), Inner Mongolia (Zheng et al. 2012).

#### Mongolotettix vittatus

(Uvarov, 1914)

CD30652A-D54E-5AB5-A98A-298037905F48

http://orthoptera.speciesfile.org/Common/basic/Taxa.aspx?TaxonNameID=1107089

Chrysochraon kaszabi Steinmann (1967) :106-120.

##### Native status

**Distribution in the natural zone**: Taiga, forest-steppe, steppe and desert steppe.

##### Distribution

**in Mongolia**: Uvs, Khuvs., A.-khang., Bulg., Sel., Tuv, Khent., S.-baat., Do., U-khang., U.-govi. Pylnov (1916):276, Steinmann (1967):108, Steinmann (1968):240, Steinmann (1971):148, Chogsomzhav (1989):93, Sergeev et al. (2019):17, Batkhuyag and Batnaran (2021):58.

**Global distribution**: Tuva, S Siberia from Tuva and Krasnoyarsk Region to Dauria, S Amur Region, Mongolia, NE China (Sergeev et al. 2019).

#### Podismopsis altaica

(Zubovski, 1900)

D05A9B94-1547-5D27-9FE5-59D1C4B373A6

http://orthoptera.speciesfile.org/Common/basic/Taxa.aspx?TaxonNameID=1106371

##### Native status

**Distribution in the natural zone**: High mountain, forest-steppe, steppe and desert steppe.

##### Distribution

**in Mongolia**: Khent. Zubovski (1900):2, Ikonnikov (1911):246, Bey-Bienko (1933):115, Günther (1971):114, Chogsomzhav (1969b):127, Chogsomzhav (1972):158, Chogsomzhav (1989):93, Altanchimeg and Nonnaizab (2013) , Sergeev et al. (2019):18, Batkhuyag and Batnaran (2021):59.

**Global distribution**: Tuva, Altai-Sayan Mts, E Kazakhstan, N Mongolia (Sergeev et al. 2019).

#### Podismopsis ussurensis

Ikonnikov, 1911

D8B8F0A1-E8CD-5261-AAD7-80EC82C964D1

http://orthoptera.speciesfile.org/Common/basic/Taxa.aspx?TaxonNameID=1106404

##### Native status

**Distribution in the natural zone**: Forest steppe.

##### Distribution

**in Mongolia**: Khent. Chogsomzhav (1972), Storozhenko and Paik (2007):187, Storozhenko et al. (2015):269, Batkhuyag and Batnaran (2021):60.

**Global distribution**: Korea (HN, PN), Russia (far east), NE China, Mongolia (Storozhenko et al. 2015).

#### Eremippus mistshenkoi

Stebaev, 1965

D4D84F07-71C1-57E6-9F81-682EE527718A

http://orthoptera.speciesfile.org/Common/basic/Taxa.aspx?TaxonNameID=1105621

##### Native status

**Distribution in the natural zone**: Steppe.

##### Distribution

**in Mongolia**: Uvs. Chogsomzhav (1969a):77, Chogsomzhav (1969b):127, Chogsomzhav (1972)1:61, Chogsomzhav (1989):92, Sergeev et al. (2019):22, Batkhuyag and Batnaran (2021):67.

**Global distribution**: Tuva, E Kazakhstan, NW Mongolia (Sergeev et al. 2019).

#### Eremippus mongolicus

Ramme, 1952

CBCDA0D5-72AC-58E1-B8A2-2F2389D3F8C1

http://orthoptera.speciesfile.org/Common/basic/Taxa.aspx?TaxonNameID=1105622

Eremippus kozlov Mistshenko and Bey-Bienko (1951):452.

##### Native status

**Distribution in the natural zone**: Steppe, desert steppe and desert.

##### Distribution

**in Mongolia**: G.-alt., B.-khong., U-khang., U.-govi. Mistshenko and Bey-Bienko (1951):452, Mistshenko (1968):490, Chogsomzhav (1968):59, Chogsomzhav (1989):92, Sergeev et al. (2019):22, Batkhuyag and Batnaran (2021):67.

**Global distribution**: Tuva, SE Kazakhstan, W Mongolia, NW China (Mistshenko and Bey-Bienko 1951), SE European Russia, Kazakhstan (except the northern part) (Sergeev et al. 2019).

#### Eremippus simplex

(Eversmann, 1859)

73ACFBA2-E9DE-55BC-822B-601DAB3B635A

http://orthoptera.speciesfile.org/Common/basic/Taxa.aspx?TaxonNameID=1105654

##### Native status

**Distribution in the natural zone**: Steppe, desert steppe and desert.

##### Distribution

**in Mongolia**: Uvs, Khovd, G.-alt. Günther (1971):115, Chogsomzhav (1974b):27, Chogsomzhav (1989):92, Sergeev (1995): 246, Sergeev et al. (2009):108, Popova et al. (2020):600, Batkhuyag and Batnaran (2021):68.

**Global distribution**: Kazakhstan, Turkmenistan, Uzbekistan, Afghanistan, Kirgizstan, Mongolia (Childebaev and Storozhenko 2001).

#### Notostaurus albicornis

(Eversmann, 1848)

E94756A3-4265-5C85-A764-EC1D86A0CA4F

http://orthoptera.speciesfile.org/common/basic/Taxa.aspx/common/editTaxon/Distribution/Taxa.aspx?TaxonNameID=1105674

##### Native status

**Distribution in the natural zone**: Steppe, desert steppe and desert.

##### Distribution

**in Mongolia**: Khovd. Günther (1971):114, Chogsomzhav (1989):92, Childebaev and Storozhenko (2001), Sergeev et al. (2009):108, Popova et al. (2020): 599, Batkhuyag and Batnaran (2021):66.

**Global distribution**: E Europe, Caucasus range, Mongolia, W Siberia, N Iran (Garai 2010).

#### Dociostaurus (Kazakia) brevicollis

(Eversmann, 1848)

4224007F-2107-53A5-AA6F-F3ECE6E1213D

http://orthoptera.speciesfile.org/Common/basic/Taxa.aspx?TaxonNameID=1105587

##### Native status

**Distribution in the natural zone**: Forest steppe, steppe, desert steppe and desert.

##### Distribution

**in Mongolia**: Tuv, Khovd. Chogsomzhav (1969b):127, Chogsomzhav (1972):161, Chogsomzhav (1974b):28, Günther (1971):114, Sergeev (1995):245, Childebaev and Storozhenko (2001):30, Sergeev et al. (2009):108, Altanchimeg and Nonnaizab (2013), Altanchimeg et al. (2013b):65, Batkhuyag and Batnaran (2021):66.

**Global distribution**: Caucasus, Transcaucasia, East Europe, Russia East and South, Kazakhstan, Kirgizstan, Mongolia, Siberia, Iran (Childebaev and Storozhenko 2001).

#### Dociostaurus (Kazakia) tarbinskyi

(Bey-Bienko, 1933)

C9A4BFA1-B732-5192-909B-1F78D514DBA8

http://orthoptera.speciesfile.org/Common/basic/Taxa.aspx?TaxonNameID=1105586

##### Native status

**Distribution in the natural zone**: Desert.

##### Distribution

**in Mongolia**: Khovd. Chogsomzhav (1969a):77, Chogsomzhav (1989):92, Childebaev and Storozhenko (2001):30, Batkhuyag and Batnaran (2021):66.

**Global distribution**: Kazakhstan and Mongolia (Childebaev and Storozhenko 2001).

#### Eclipophleps bogdanovi

Tarbinsky, 1927

B5BA7AE8-B1A8-5BBE-A24D-F24B6B6942F5

http://orthoptera.speciesfile.org/Common/basic/Taxa.aspx?TaxonNameID=1105104

##### Native status

**Distribution in the natural zone**: High mountain, steppe and desert steppe.

##### Distribution

**in Mongolia**: Altai. Tarbinsky (1927):495, Günther (1971):123, Chogsomzhav (1972):175, Batkhuyag (1995):27, Chogsomzhav (1989):91, Sergeev et al. (2009):108, Altanchimeg (2011):16, Altanchimeg and Nonnaizab (2013), Batnaran et al. (2016):35, Batkhuyag and Batnaran (2021):61.

#### Eclipophleps carinata

Mistshenko, 1968

1DE0DF66-8793-5B01-B9E4-79F249C5AFDD

http://orthoptera.speciesfile.org/Common/basic/Taxa.aspx?TaxonNameID=1105105

##### Native status

**Distribution in the natural zone**: High mountain, desert steppe and desert.

##### Distribution

**in Mongolia**: B.-khong., G.-alt. Mistshenko (1968):493, Steinmann (1968):245, Sergeev (1995):243, Qian et al. (2021):1310, Batkhuyag and Batnaran (2021):63.

#### Eclipophleps confinis

Mistshenko, 1951

0B70F117-5056-51B5-AF6D-492E9AD21B89

http://orthoptera.speciesfile.org/Common/basic/Taxa.aspx?TaxonNameID=1105116

Oreoptygonotus mongolicus Steinmann (1968):245.

##### Native status

**Distribution in the natural zone**: High mountain, steppe and desert steppe.

##### Distribution

**in Mongolia**: Khovd, G.-alt., B.-khong, U.-govi., U-khang. Günther (1971):123, Mistshenko (1968):495, Chogsomzhav (1972):175, Chogsomzhav (1989):91, Sergeev (1995):243, Sergeev et al. (2009):108, Altanchimeg (2011):16, Batnaran et al. (2016):35, Batkhuyag and Batnaran (2021):63.

#### Eclipophleps glacialis

Bey-Bienko, 1933

FD43FAA7-E689-5512-84B3-526547447B8B

http://orthoptera.speciesfile.org/Common/basic/Taxa.aspx?TaxonNameID=1105106

##### Native status

**Distribution in the natural zone**: High mountain, steppe and desert steppe.

##### Distribution

**in Mongolia**: B.-Ulg., Uvs. Bey-Bienko (1933):115, Chogsomzhav (1969b):128, Chogsomzhav (1989):91, Batkhuyag (1995):27, Sergeev et al. (2009):108, Altanchimeg (2011):16, Sergeev et al. (2019):19, Batkhuyag and Batnaran (2021):62.

#### Eclipophleps kerzhneri

Mistshenko, 1968

B3977920-A8CA-5262-B81D-D23829CA3B04

http://orthoptera.speciesfile.org/Common/basic/Taxa.aspx?TaxonNameID=1105108

##### Native status

**Distribution in the natural zone**: High mountain, steppe, desert steppe and desert.

##### Distribution

**in Mongolia**: G.-alt. Mistshenko (1968):493, Batkhuyag (1995):28, Altanchimeg (2011):16, Batkhuyag and Batnaran (2021):62.

#### Eclipophleps lucida

Mistshenko, 1973

1C881501-0260-5D0E-8589-0504904B1D19

http://orthoptera.speciesfile.org/Common/basic/Taxa.aspx?TaxonNameID=1105110

##### Native status

**Distribution in the natural zone**: High mountain, steppe, desert steppe and desert.

##### Distribution

**in Mongolia**: Uvs, Khovd. Mistshenko (1973), Chogsomzhav (1977):86, Altanchimeg (2011):16, Batkhuyag and Batnaran (2021):62.

#### Eclipophleps similis

Mistshenko, 1951

4F0E26C5-659C-5444-AD2A-049C2C7172B9

http://orthoptera.speciesfile.org/Common/basic/Taxa.aspx?TaxonNameID=1105111

##### Native status

**Distribution in the natural zone**: High mountain, steppe, desert steppe and desert.

##### Distribution

**in Mongolia**: Uvs, Khovd, B.-Ulg., Tuv. Mistshenko and Bey-Bienko (1951):549, Steinmann (1968):243, Batkhuyag (1995):27, Sergeev et al. (2009):108, Altanchimeg (2011):16, Batkhuyag and Batnaran (2021):61.

#### Eclipophleps tarbinskii

Orishchenko, 1960

9A325411-544F-554C-8FFD-EC412555C99E

http://orthoptera.speciesfile.org/Common/basic/Taxa.aspx?TaxonNameID=1105112

##### Native status

**Distribution in the natural zone**: High mountain, steppe and desert steppe.

##### Distribution

**in Mongolia**: Altai. Orishchenko. (1960), Chogsomzhav (1989):91, Batkhuyag (1995):27, Sergeev et al. (2009):108, Altanchimeg (2011):16, Altanchimeg and Nonnaizab (2013), Batkhuyag and Batnaran (2021):61.

#### Chorthippus (Altichorthippus) intermedius

(Bey-Bienko, 1926)

E6E05A34-BE45-5013-8DC8-B48005741443

http://orthoptera.speciesfile.org/Common/basic/Taxa.aspx?TaxonNameID=1106089

##### Native status

**Distribution in the natural zone**: Taiga, forest-steppe and steppe.

##### Distribution

**in Mongolia**: Uvs, Khuvs., Bulg., Sel., Tuv, Khent., S.-baat., Do., U-khang., A.-khang. Tarbinsky (1927):492, Cejchan and Maran (1966):183, Mistshenko (1968):492, Steinmann (1967):116-117, Steinmann (1968):243, Chogsomzhav (1969b):127, Storozhenko and Paik (2007), Altanchimeg et al. (2013b):65, Batkhuyag and Batnaran (2021):87.

**Global distribution**: Tuva, Altai-Sayan Mts, Sakha (Yakutia), S Russian Far East (including Sakhalin), Mongolia N, NE China, Tibet (Sergeev et al. 2020).

#### Chorthippus (Chorthippus) albomarginatus

(De Geer, 1773)

4A6EEBB4-6CDE-588B-901B-61C76FA30627

http://orthoptera.speciesfile.org/Common/basic/Taxa.aspx?TaxonNameID=1105771

##### Native status

**Distribution in the natural zone**: Taiga, forest-steppe, steppe, desert steppe and desert

##### Distribution

**in Mongolia**: Uvs, Zav., Bulg., Khovd. Bey-Bienko (1933):115, Chogsomzhav (1969b):128, Chogsomzhav 1972:173, Günther 1971:123, Chogsomzhav 1989:92, Sergeev 1995:251, Sergeev et al. (2009):109, Altanchimeg and Nonnaizab (2013) Batnaran et al. (2016):14-15, Sergeev et al. (2020):10, Batkhuyag and Batnaran (2021):87.

**Global distribution**: South Korea (Storozhenko et al. 2015) Tuva, Europe (except the extreme north and the southern parts), W Siberia, N Kazakhstan, N Mongolia (Sergeev et al. 2020).

#### Chorthippus (Chorthippus) caliginosus

Mistshenko, 1951

4FCF6C04-3285-5E11-A5CF-A8096985249B

http://orthoptera.speciesfile.org/Common/basic/Taxa.aspx?TaxonNameID=1105762

##### Native status

**Distribution in the natural zone**: Forest steppe.

##### Distribution

**in Mongolia**: Sel. Garai (2001):751, Batkhuyag and Batnaran (2021):87.

**Global distribution**: Southern regions of Transbaikalia, Amur area, Khabarovsk region and south-east of China (Tishechkin and Bukhvalova 2009), Mongolia Garai (2001).

#### Chorthippus (Chorthippus) dorsatus

(Zetterstedt, 1821)

BE231203-5710-5C3E-83FB-D8E2A260B569

http://orthoptera.speciesfile.org/Common/basic/Taxa.aspx?TaxonNameID=1105795

##### Native status

**Distribution in the natural zone**: Forest steppe, steppe and desert steppe.

##### Distribution

**in Mongolia**: Uvs, S.-baat. Chogsomzhav (1969b):128, Chogsomzhav (1972):173, Sergeev et al. (2020):8, Batkhuyag and Batnaran (2021):86.

**Global distribution**: Tuva, Transbaikalia, Mongolia, NE China (Sergeev et al. 2020).

#### Chorthippus (Chorthippus) hammarstroemi

(Miram, 1907)

9CCD24C3-42BC-586A-B61B-195E56F5924A

http://orthoptera.speciesfile.org/Common/basic/Taxa.aspx?TaxonNameID=1105913

##### Native status

**Distribution in the natural zone**: Taiga, forest-steppe, steppe, desert steppe and desert.

##### Distribution

**in Mongolia**: Bulg., A.-khang., Sel., Tuv, S.-baat., Khuvs. Cejchan and Maran (1966):183, Chogsomzhav (1971):80, Sergeev (1995):249, Storozhenko and Paik (2007):173, Storozhenko et al. (2015):242, Altanchimeg and Nonnaizab (2013), Altanchimeg et al. (2013b):65, Batnaran et al. (2016):34, Sergeev et al. (2020):7, Batkhuyag and Batnaran (2021):83.

**Global distribution**: South Korea (Storozhenko et al. 2015), Tuva, Altai-Sayan Mts, Transbaikalia, S Sakha (Yakutia), S Russian Far East, Mongolia, N, NE China (Sergeev et al. 2020).

#### Chorthippus (Chorthippus) dichrous

(Eversmann, 1859)

953D4E74-9252-5876-894C-F11228C40085

http://orthoptera.speciesfile.org/Common/basic/Taxa.aspx?TaxonNameID=1105796

Chorthippus dorsatus australia Predtechenskii (1928):89.

##### Native status

**Distribution in the natural zone**: High mountain, forest-steppe, steppe, desert steppe and desert.

##### Distribution

**in Mongolia**: B.-Ulg., S.-baat., Do., B.-khong., Sel., Khovd. Pylnov (1916):278, Chogsomzhav (1971):82, Chogsomzhav (1972):173, Sergeev (1995):251, Childebaev and Storozhenko (2001), Sergeev et al. (2009):109, Batnaran et al. (2016):33, Sergeev et al. (2020):9, Batkhuyag and Batnaran (2021):86.

**Global distribution**: Tuva, S, SE Europe, S Siberia (up to Tuva), Asia Minor, Caucasus, Iran, Kazakhstan, Tien Shan, Pamiro-Alay, NW China, Mongolia (Sergeev et al. 2020).

#### Chorthippus (Chorthippus) fallax

(Zubovski, 1900)

DD4E4ABF-C80E-54A2-8924-E6C7945AF136

http://orthoptera.speciesfile.org/Common/basic/Taxa.aspx?TaxonNameID=1105903

##### Native status

**Distribution in the natural zone**: Taiga, forest-steppe, steppe and desert steppe.

##### Distribution

**in Mongolia**: B.-Ulg., Uvs, Zav., Khuvs., A.-khang., Bulg., Sel., Tuv, Khent., S.-baat., Da., G.-alt., B.-khong. Ikonnikov (1911):253, Pylnov (1916):278, Bey-Bienko (1933):115, Cejchan and Maran (1966):183, Mistshenko (1968):492, Günther (1971):122, Chogsomzhav (1968):57, Chogsomzhav (1969b):128, Chogsomzhav (1972):172, Munkhbat (2010):16, Altanchimeg and Nonnaizab (2013), Altanchimeg et al. (2013b):65, Storozhenko et al. (2015):244, Sergeev et al. (2020):85.

**Global distribution**: Tuva, Siberia (except the western part of West Siberian Plain and the extreme north), S Russian Far East, E Kazakhstan, N Mongolia, N, NE China, South Korea (Storozhenko et al. 2015, Sergeev et al. 2020).

#### Chorthippus (Chorthippus) turanicus

Tarbinsky, 1925

3C651F0D-0524-5C15-B208-1B7E12D88109

http://orthoptera.speciesfile.org/Common/basic/Taxa.aspx?TaxonNameID=1105894

##### Native status

**Distribution in the natural zone**: Steppe.

##### Distribution

**in Mongolia**: G.-alt. Chogsomzhav (1974b):29, Chogsomzhav (1989):92, Sergeev et al. (2009):109, Batkhuyag and Batnaran (2021):84.

**Global distribution**: China, Xinjiang (Tarbinsky 1925), Turkestan, Kazakhstan, Tadzhikistan, Uzbekistan (Childebaev and Storozhenko 2001), Mongolia (Sergeev et al. 2009).

#### Chorthippus (Chorthippus) ilkazi

Uvarov, 1934

688AB4E6-447D-5D42-B995-944E2327B937

http://orthoptera.speciesfile.org/Common/basic/Taxa.aspx?TaxonNameID=1105936

##### Native status

**Distribution in the natural zone**: Steppe and desert steppe.

##### Distribution

**in Mongolia**: Tuv, S.-baat., Do. Steinmann (1967):116, Altanchimeg et al. (2013b):65.

**Global distribution**: Western Asia, Mongolia, Turkey (Uvarov 1934).

#### Chorthippus (Glyptobothrus) apricarius

(Linnaeus, 1758)

361B4E0A-54A0-5D15-A7EB-BCF7666DF9C5

http://orthoptera.speciesfile.org/Common/basic/Taxa.aspx?TaxonNameID=1105949

##### Native status

**Distribution in the natural zone**: Forest steppe and steppe.

##### Distribution

**in Mongolia**: Uvs, Bulg., Sel., Tuv, Khent., U-khang. Pylnov (1916):278, Steinmann (1967):114, Chogsomzhav (1968):57-58, Chogsomzhav (1969b):127, Chogsomzhav (1989):92, Childebaev and Storozhenko (2001):34, Sergeev et al. (2009):108, Altanchimeg and Nonnaizab (2013), Sergeev et al. (2020):6, Batkhuyag and Batnaran (2021):82.

**Global distribution**: Tuva, Europe (except the extreme north), S Siberia, Asia Minor, Kazakhstan, NW, N, NE China, Mongolia (Sergeev et al. 2020).

#### Chorthippus (Glyptobothrus) biguttulus

(Linnaeus, 1758)

8D258899-874F-593E-B45B-267BE49D0200

http://orthoptera.speciesfile.org/Common/basic/Taxa.aspx?TaxonNameID=1106017

##### Native status

**Distribution in the natural zone**: Taiga, forest-steppe, steppe and desert steppe.

##### Distribution

**in Mongolia**: Uvs, Bulg., Sel., Tuv, Khent., U-khang. Pylnov (1916):278, Bey-Bienko (1933):115, Steinmann (1967):115, Mistshenko (1968):492, Steinmann (1968):243, Chogsomzhav (1969b):127, Chogsomzhav (1974b):28, Sergeev (1995):248, Sergeev et al. (2009):108, Altanchimeg and Nonnaizab (2013), Myagmar et al. (2019):56, Batkhuyag and Batnaran (2021):79.

**Global distribution**: France, Switzerland, Yugoslavia (Miksic 1981), Mongolia (Steinmann 1968), Inner Mongolia (Li et al. 2007).

#### Chorthippus (Glyptobothrus) brunneus

(Thunberg, 1815)

A881A741-AC9E-5216-924E-C641FFEFC75D

http://orthoptera.speciesfile.org/Common/basic/Taxa.aspx?TaxonNameID=1106050

##### Native status

**Distribution in the natural zone**: Taiga, forest-steppe, steppe and desert steppe.

##### Distribution

**in Mongolia**: B.-Ulg., Uvs, Khuvs., Bulg., Tuv, S.-baat., Do., Khovd., B.-khong., Du.-govi. Bolívar (1901):226, Bey-Bienko (1933):115, Steinmann (1968):243, Chogsomzhav (1970), Chogsomzhav (1989):92, Childebaev and Storozhenko (2001), Altanchimeg et al. (2013b):65, Batnaran et al. (2016):33, Sergeev et al. (2019):34, Batkhuyag and Batnaran (2021):78.

**Global distribution**: Tuva, S Russia up to Tuva, N Kazakhstan, N Mongolia (Benediktov 1999, Sergeev et al. 2019).

#### Chorthippus (Glyptobothrus) maritimus

Mistshenko, 1951

AB6FF0B4-1F8C-5755-942B-A91D1D61B068

http://orthoptera.speciesfile.org/Common/basic/Taxa.aspx?TaxonNameID=1106041

##### Native status

**Distribution in the natural zone**: High mountain and desert steppe.

##### Distribution

**in Mongolia: Altanchimeg and Nonnaizab (2013)**:81.

**Global distribution**: Caucasus, Transcaucasus, East Europe, Krym and China (Ramme 1939), Mongolia (**Altanchimeg and Nonnaizab 2013)**.

#### Chorthippus (Glyptobothrus) dubius

(Zubovski, 1898)

52445A4E-3207-503C-92B7-20FF96E66D94

http://orthoptera.speciesfile.org/Common/basic/Taxa.aspx?TaxonNameID=1105932

##### Native status

**Distribution in the natural zone**: Forest steppe, steppe and desert steppe.

##### Distribution

**in Mongolia**: B.-Ulg., Uvs, Zav., Khuvs., A.-khang., Bulg., Sel., Tuv, Khent., Do., G.-alt., B.-khong., U-khang., U.-govi. Bolívar (1901):226, 231, Bey-Bienko (1933):115, Steinmann (1967):116, Mistshenko (1968):492, Chogsomzhav (1968):58, Chogsomzhav (1969b):128, Chogsomzhav (1972):171, Günther (1971):121, Sergeev (1995):248, Childebaev and Storozhenko (2001), Altanchimeg and Nonnaizab (2013), Altanchimeg et al. (2013b):65, Batnaran et al. (2016):34, Sergeev et al. (2019):38, Batkhuyag and Batnaran (2021):81.

**Global distribution**: Tuva, SE European Russia, S Siberia, Kazakhstan, Mongolia, NE, N, C China (Sergeev et al. 2019).

#### Chorthippus (Glyptobothrus) mollis

(Charpentier, 1825)

7B007093-BDAA-5F6D-800E-82EEDA1D7552

http://orthoptera.speciesfile.org/Common/basic/Taxa.aspx?TaxonNameID=1106033

##### Native status

**Distribution in the natural zone**: Desert steppe and desert.

##### Distribution

**in Mongolia**: Altanchimeg and Nonnaizab (2013):81, Batkhuyag and Batnaran (2021):80.

**Global distribution**: Tuva, Europe (except the extreme north), Siberia (except the extreme north), Asia Minor, Caucasus, Kazakhstan, Middle Asia, N Iran (Sergeev et al. 2019), Mongolia (Altanchimeg and Nonnaizab 2013).

#### Chorthippus (Glyptobothrus) vagans

(Eversmann, 1848)

BE3DAD8D-0E6F-5716-A35D-C2006A5BAF14

http://orthoptera.speciesfile.org/Common/basic/Taxa.aspx?TaxonNameID=1105971

Gomphocerus subsinuatus Fischer (1849):42.

##### Native status

**Distribution in the natural zone**: Forest steppe.

##### Distribution

**in Mongolia**: Altanchimeg and Nonnaizab (2013):81, Batkhuyag and Batnaran (2021):87.

**Global distribution**: from West Europe far into temperate Asia (Willemse 2009), Mongolia (Altanchimeg and Nonnaizab 2013).

#### Pseudochorthippus montanus

(Charpentier, 1825)

C5B43D4C-8802-53BA-9659-DBFE6B4FD681

http://orthoptera.speciesfile.org/Common/basic/Taxa.aspx?TaxonNameID=1106350

Stenobothrus (Chorthippus) longicornis Jacobson and Bianchi. (1905):182,234.
Chorthippus longicornis Jacobson and Bianchi. (1905):182.

##### Native status

**Distribution in the natural zone**: Taiga, forest-steppe, steppe, desert steppe and desert.

##### Distribution

**in Mongolia**: B.-Ulg., Uvs, Zav., A.-khang., Bulg., Tuv, B.-khong., Khovd. Mistshenko (1968):492, Chogsomzhav (1969b):127, Chogsomzhav (1972):173, Günther (1971):122, Sergeev (1995):251, Childebaev and Storozhenko (2001), Sergeev et al. (2009):109, Altanchimeg et al. (2013b):65, Storozhenko et al. (2015):246, Sergeev et al. (2020):15, Batkhuyag and Batnaran (2021):85.

**Global distribution**: South Korea (Storozhenko et al. 2015), Tuva, N, C Europe, Siberia, Russian Far East, N Kazakhstan, Mongolia, NE China, N Korea (Sergeev et al. 2020).

#### Pseudochorthippus parallelus

(Zetterstedt, 1821)

59BCE02F-56AF-5C45-975F-CBB2E7BDE7D0

http://orthoptera.speciesfile.org/Common/basic/Taxa.aspx?TaxonNameID=1106327

##### Native status

**Distribution in the natural zone**: Steppe.

##### Distribution

**in Mongolia**: Uvs. Chogsomzhav (1977), Batkhuyag and Batnaran (2021):85.

**Global distribution**: Tuva, Europe (except the extreme north), Siberia (except the north, but including the central parts of Sakha (Yakutia) and the southern parts of Krasnoyarsk Region and the Republic of Khakassia (Ivanova 1967), Asia Minor, Caucasus, Kazakhstan, Tien Shan, Mongolia [including the Mongolian part of Uvs-Nuur Basin (Chogsomzhav 1977), NW China (Sergeev et al. 2020).

#### Aeropedellus baliolus

Mistshenko, 1951

CE35E01C-E98D-5AAD-BF0C-CE188F5BF7FB

http://orthoptera.speciesfile.org/Common/basic/Taxa.aspx?TaxonNameID=1106225

##### Native status

**Distribution in the natural zone**: Steppe.

##### Distribution

**in Mongolia**: Tuv, S.-baat., U-khang. Steinmann (1967):114, Altanchimeg et al. (2014):133, Popova et al. (2020):602.

**Global distribution**: Kazakhstan and Mongolia (Altanchimeg et al. 2014).

#### Aeropedellus chogsomjavi

Altanchimeg, Chen & Nonnaitzb, 2014

F92C17AE-CF4B-5B36-87E5-0D1D61896D70

http://orthoptera.speciesfile.org/Common/basic/Taxa.aspx?TaxonNameID=1220729

##### Native status

**Distribution in the natural zone**: Steppe.

##### Distribution

**in Mongolia**: Khuvs. Altanchimeg and Nonnaizab (2013):81, Altanchimeg et al. (2014):133, Batkhuyag and Batnaran (2021):76.

#### Aeropedellus reuteri

(Miram, 1907)

EA0595DC-DACB-5493-8B89-7768FAC5AF45

http://orthoptera.speciesfile.org/Common/basic/Taxa.aspx?TaxonNameID=1106234

##### Native status

**Distribution in the natural zone**: Steppe and desert steppe.

##### Distribution

**in Mongolia**: Khent., U-khang. Steinmann (1967):114, Altanchimeg et al. (2014):133, Sergeev et al. (2020):4, Batkhuyag and Batnaran (2021):76.

**Global distribution**: Mongolia, Type localities and Khakassia (Sergeev et al. 2020).

#### Aeropedellus variegatus

(Fischer von Waldheim, 1846)

696B5033-C52F-5B6B-B087-F2474BB8CC3E

http://orthoptera.speciesfile.org/Common/basic/Taxa.aspx?TaxonNameID=1106239

##### Native status

**Distribution in the natural zone**: Forest steppe and steppe.

##### Distribution

**in Mongolia**: B.-Ulg, Uvs, Zav., Khuvs., A.-khang, Bulg., Tuv., Khent., Khovd, G.-alt., B.-khong., U-khang. Cejchan and Maran (1966):182, Steinmann (1968):242, Steinmann (1967):113, Chogsomzhav (1969b):127, Günther (1971):120,Sergeev (1995):247, Sergeev et al. (2009):108, Altanchimeg (2011):16, Altanchimeg and Nonnaizab (2013), Altanchimeg et al. (2013b):65, Altanchimeg et al. (2014):133, Sergeev et al. (2020):4, Batkhuyag and Batnaran (2021):75.

**Global distribution**: Tuva, N Caucasus, NE European Russia, Siberia, N Europe, mountains of S Europe, E Kazakhstan, Mongolia (Sergeev et al. 2020).

#### Gomphocerus sibiricus

(Linnaeus, 1767)

CDE709EA-AADC-5C18-9C27-523CF76F9173

http://orthoptera.speciesfile.org/Common/basic/Taxa.aspx?TaxonNameID=1106148&Next=Taxa.aspx

##### Native status

**Distribution in the natural zone**: Forest steppe and steppe.

##### Distribution

**in Mongolia**: Uvs, Zav., Khuvs., A.-khang., Bulg., Tuv, Khent., Khovd, G.-alt., U-khang., U.-govi., Cejchan and Maran (1966):180, Günther (1971):119, Mistshenko (1968):492, Chogsomzhav (1989):91, Sergeev et al. (2009):108, Altanchimeg and Nonnaizab (2013), Altanchimeg et al. (2013b):65, Batkhuyag and Batnaran (2021):74.

**Global distribution**: Tuva, N, NE Europe, Siberia (except the extreme north), N Kazakhstan, N Mongolia, NE China (Sergeev et al. 2020).

#### Gomphocerippus rufus

(Linnaeus, 1758)

88AF9AC6-A151-5F23-BFC4-C0FCAEE99754

http://orthoptera.speciesfile.org/Common/basic/Taxa.aspx?TaxonNameID=1106254

Acrydium clavicorne De Geer (1773):482.

##### Native status

**Distribution in the natural zone**: Forest steppe and steppe.

##### Distribution

**in Mongolia**: Tuv. Childebaev and Storozhenko (2001), Munkhbat (2010):168, Batkhuyag and Batnaran (2021):73.

**Global distribution**: Mongolia (Childebaev and Storozhenko 2001), Tuva, Europe (except the extreme north), Siberia (except the extreme north and NE parts), Amur Region, N Caucasus, W Kazakhstan, NE China (Sergeev et al. 2020).

#### Myrmeleotettix palpalis

(Zubovski, 1900)

7CCFEA98-0456-5DBC-88E1-0E38B3EE7ECB

http://orthoptera.speciesfile.org/Common/basic/Taxa.aspx?TaxonNameID=1106193

##### Native status

**Distribution in the natural zone**: Forest steppe, steppe, desert steppe and desert.

##### Distribution

**in Mongolia**: B.-Ulg., Uvs, Zav., Khuvs., Khovd, G.-alt., Sel., Tuv., Khent., A.-khang., U-khang., U.-govi. Zubovski (1900):13, Pylnov (1916):278, Bey-Bienko (1933):118, Cejchan and Maran (1966):180, Chogsomzhav (1968):56-58, Chogsomzhav (1969b):127, Chogsomzhav (1974b):28, Chogsomzhav (1989):91, Childebaev and Storozhenko (2001), Garai (2001):751, Sergeev et al. (2009):108, Altanchimeg (2011):16, Altanchimeg and Nonnaizab (2013), Altanchimeg et al. (2013b):65, Sergeev et al. (2019):29, Batkhuyag and Batnaran (2021):72.

**Global distribution**: Tuva, S Siberia (from the Altai Mts. to Dauria), Amur Region, E Kazakhstan, Mongolia, China (Sergeev et al. 2019).

#### Myrmeleotettix zaitzevi

Mistshenko, 1968

7F1D5B06-B502-541B-995E-92E549041F60

http://orthoptera.speciesfile.org/Common/basic/Taxa.aspx?TaxonNameID=1106195

##### Native status

**Distribution in the natural zone**: Desert steppe.

##### Distribution

**in Mongolia**: Du.-govi., Tuv. Mistshenko (1968):490, Chogsomzhav (1989):91, Munkhbat (2010):168, Batkhuyag and Batnaran (2021):72.

#### Stauroderus scalaris

(Fischer von Waldheim, 1846)

9C142574-B0BB-5A0A-B87E-493DA30A328D

http://orthoptera.speciesfile.org/Common/basic/Taxa.aspx?TaxonNameID=1106274

##### Native status

**Distribution in the natural zone**: Forest steppe and steppe.

##### Distribution

**in Mongolia**: Uvs, Bulg., Tuv. Pylnov (1916):278, Cejchan and Maran (1966):184, Steinmann (1964):382, Günther (1971):121, Chogsomzhav (1969b):127, Chogsomzhav (1972):168, Chogsomzhav (1989):92, Sergeev et al. (2009):108, Altanchimeg and Nonnaizab (2013), Altanchimeg et al. (2013b):65, Batnaran et al. (2016):35, Sergeev et al. (2019):32, Batkhuyag and Batnaran (2021):77.

**Global distribution**: Tuva, Europe (except the north), S Siberia (up to Buryatia), Asia Minor, Caucasus, Kazakhstan, Tien Shan, Pamiro-Alay, NW China, Mongolia, NW Iran (Sergeev et al. 2019).

#### Schmidtiacris schmidti

(Ikonnikov, 1913)

0BEA0C7C-5FFB-57ED-BBFB-643EADE682CB

http://orthoptera.speciesfile.org/Common/basic/Taxa.aspx?TaxonNameID=1114808

Chorthippus nakazimai Furukawa et al. (1950):30.

##### Native status

**Distribution in the natural zone**: Forest steppe.

##### Distribution

**in Mongolia**: Bulg., Tuv. Mistshenko (1968) :492, Günther (1971):122, Altanchimeg et al. (2013b):65, Sergeev et al. (2019):39, Batkhuyag and Batnaran (2021):83.

**Global distribution**: Tuva, Transbaikalia, S Russian Far East, Mongolia, NE China, Korea, Japan (Sergeev et al. 2019).

#### Mesasippus kozhevnikovi

(Tarbinsky, 1925)

4DFB78BC-2CBD-5DA3-8679-87E1E3E8F50B

http://orthoptera.speciesfile.org/Common/basic/Taxa.aspx?TaxonNameID=1106179

##### Native status

**Distribution in the natural zone**: Desert steppe and desert.

##### Distribution

**in Mongolia**: B.-Ulg., Uvs, Zav., Khuvs., Tuv, A.-khang., Bulg., Tuv., Sel., Khent., S.-baat., Khovd, U-khang. Mistshenko and Bey-Bienko (1951):501, Mistshenko (1968):492, Steinmann (1968):240, 242, Chogsomzhav (1972):168, Chogsomzhav (1974b):28, Chogsomzhav (1989):91, Gorochov et al. (1989):104, Childebaev and Storozhenko (2001), Batkhuyag and Batnaran (2021):77.

**Global distribution**: China, Xinjiang, Mongolia, Kazakhstan (Mistshenko and Bey-Bienko 1951, Childebaev and Storozhenko 2001).

#### Dasyhippus barbipes

(Fischer von Waldheim, 1846)

4F96FC7B-D3E3-5EE8-8CC6-42399FC9146C

http://orthoptera.speciesfile.org/Common/basic/Taxa.aspx?TaxonNameID=1106266

##### Native status

**Distribution in the natural zone**: Steppe, desert steppe and desert.

##### Distribution

**in Mongolia**: Khuvs., Sel., Tuv, S.-baat., Do., Khovd, Du.-govi. Günther (1971):120, Steinmann (1967):113, Pylnov (1916):278, Tarbinsky (1931):140, Chogsomzhav (1968):58, Chogsomzhav (1971):71, Chogsomzhav (1972):166, Chogsomzhav (1989):91, Altanchimeg and Nonnaizab (2013), Altanchimeg et al. (2013b):65, Batnaran et al. (2016):34, Sergeev et al. (2020):5, Batkhuyag and Batnaran (2021):75.

**Global distribution**: Tuva, SE Altai, Transbaikalia, Mongolia, N China (Sergeev et al. 2020).

#### Egnatioides desertus

Uvarov, 1926

6755E1E2-B6D0-52C6-B2EB-A352E2925F5A

http://orthoptera.speciesfile.org/Common/basic/Taxa.aspx?TaxonNameID=1109695

##### Native status

**Distribution in the natural zone**: Desert.

##### Distribution

**in Mongolia**: Khovd, Altai Transaltai gobi. Günther (1971):113, Chogsomzhav (1989):90, Batkhuyag and Batnaran (2021):47.

**Global distribution**: Mongolia (Günther 1971), Kazakhstan and Turkestan (Childebaev and Storozhenko 2001).

#### Omocestus (Omocestus) haemorrhoidalis

(Charpentier, 1825)

C8975DCD-53C0-5F85-96F4-4A26FA379953

http://orthoptera.speciesfile.org/Common/basic/Taxa.aspx?TaxonNameID=1107287

##### Native status

**Distribution in the natural zone**: Forest steppe and steppe.

##### Distribution

**in Mongolia**: Zav., Bulg., Tuv, Khent., S.-baat., Khovd, U-khang., Sel., Uvs, A.-khang., Khuvs. Pylnov (1916):277, Bey-Bienko (1933):118, Cejchan and Maran (1966):180, Steinmann (1967):110, Steinmann (1968):241, Chogsomzhav (1968):58, Chogsomzhav (1969b):127, Chogsomzhav (1974b):28, Sergeev (1995):246, Sergeev et al. (2009):108, Altanchimeg and Nonnaizab (2013), Altanchimeg et al. (2013b):65, Storozhenko et al. (2015):254, Sergeev et al. (2019):26, Batkhuyag and Batnaran (2021):71.

**Global distribution**: Tuva, Europe (except the extreme north), Siberia (except the extreme north), S Russian Far East, Asia Minor, Caucasus, Kazakhstan, Tien Shan, Pamiro-Alay, Mongolia, N China, South Korea (Sergeev et al. 2019).

#### Omocestus (Omocestus) petraeus

(Brisout de Barneville, 1856)

A832F6F9-C74F-5CCA-8577-742E1989D787

http://orthoptera.speciesfile.org/Common/basic/Taxa.aspx?TaxonNameID=1107268

##### Native status

**Distribution in the natural zone**: Steppe.

##### Distribution

**in Mongolia**: Uvs, Bulg., Tuv, Khent., U-khang. Steinmann (1964):382, Steinmann (1967):110, Chogsomzhav (1969b):127, Chogsomzhav (1971):66, Chogsomzhav (1972):164, Batnaran et al. (2016):35, Sergeev et al. (2020):30, 32, Batkhuyag and Batnaran (2021):71.

**Global distribution**: Tuva, S Europe, S Siberia (up to S Krasnoyarsk Region), Asia Minor, Caucasus, N Kazakhstan (Sergeev et al. 2019), Mongolia (Sergeev et al. 2020).

#### Omocestus (Omocestus) rufipes

(Zetterstedt, 1821)

0621189A-53ED-5376-8EBE-46B8D8B8A84C

http://orthoptera.speciesfile.org/Common/basic/Taxa.aspx?TaxonNameID=1107273

##### Native status

**Distribution in the natural zone**: Steppe.

##### Distribution

**in Mongolia**: Chogsomzhav (1969b):91, Childebaev and Storozhenko (2001):32, Altanchimeg and Nonnaizab (2013).

**Global distribution**: Russian C, E, S, Kazakhstan, Siberia, Mongolia (Childebaev and Storozhenko 2001).

#### Omocestus (Omocestus) tzendsureni

Günther, 1971

C567F9FA-56E2-5200-A861-971F68C33D01

http://orthoptera.speciesfile.org/Common/basic/Taxa.aspx?TaxonNameID=1107282

##### Native status

**Distribution in the natural zone**: Forest steppe, steppe and desert steppe.

##### Distribution

**in Mongolia**: Khovd, G.-alt. Günther (1971):116, Chogsomzhav (1989):91, Sergeev (1995):246, Batkhuyag and Batnaran (2021):71.

**Global distribution**: China, Xinjiang, Mongolia (Günther 1971).

#### Omocestus (Omocestus) viridulus

(Linnaeus, 1758)

3B153922-A93A-5A92-821E-E07EF67C8656

http://orthoptera.speciesfile.org/Common/basic/Taxa.aspx?TaxonNameID=1107300

##### Native status

**Distribution in the natural zone**: Forest steppe, steppe, desert steppe and desert.

##### Distribution

**in Mongolia**: Khuvs., Bulg., Sel., S.-baat., Do., Khovd, G.-alt., Uvs. Steinmann (1964):382, Steinmann (1967):110, Steinmann (1968):241, Chogsomzhav (1968):57, Chogsomzhav (1969b):127, Chogsomzhav (1972):163, Chogsomzhav (1974b):28, Günther (1971):116, Chogsomzhav (1989):91, Sergeev (1995):108, Childebaev and Storozhenko (2001), Sergeev et al. (2009):108, Altanchimeg and Nonnaizab (2013), Altanchimeg et al. (2013b):65, Batnaran et al. (2016):36, Sergeev et al. (2019):26, Batkhuyag and Batnaran (2021):70.

**Global distribution**: Tuva, Europe (except the extreme north), S Siberia, Amur Region, S Khabarovsk Region, Asia Minor, Caucasus, Kazakhstan, Tien Shan, N China, Mongolia, N Korea (Sergeev et al. 2019).

#### Stenobothrus carbonarius

(Eversmann, 1848)

385404C5-AC1F-5D54-B968-2C64B2BF5092

http://orthoptera.speciesfile.org/common/basic/Taxa.aspx/common/Links/Taxa.aspx?TaxonNameID=1107430

##### Native status

**Distribution in the natural zone**: Forest steppe.

##### Distribution

**in Mongolia**: Sel. Sergeev (1995):246, Childebaev and Storozhenko (2001):31, Batnaran (2008):47, Batnaran et al. (2016):36, Popova et al. (2020):600, Batkhuyag and Batnaran (2021):69.

**Global distribution**: Mongolia (Childebaev and Storozhenko 2001), Tuva, SE European Russia, S Siberia (up to Buryatia), Kazakhstan (Sergeev et al. 2019).

#### Stenobothrus newskii

Zubovski, 1900

E54CA99F-0E89-50F3-AF1A-B4126580CED7

http://orthoptera.speciesfile.org/common/basic/Taxa.aspx/common/Links/Taxa.aspx?TaxonNameID=1107354

##### Native status

**Distribution in the natural zone**: Taiga and forest-steppe.

##### Distribution

**in Mongolia**: G.-alt. Zubovski (1900):9, Bey-Bienko (1926):10, Chogsomzhav (1972):162, Sergeev et al. (2009):108, Altanchimeg (2011):16, Sergeev et al. (2019):24, Batkhuyag and Batnaran (2021):70.

**Global distribution**: Tuva, Altai Mts. (including S Altai), NW Mongolia (Sergeev et al. 2019).

#### Stenobothrus eurasius

Zubovski, 1898

EEC48FE2-A45E-516B-9D02-BCE0CE38DB70

http://orthoptera.speciesfile.org/common/basic/Taxa.aspx/common/Links/Taxa.aspx?TaxonNameID=1107386

##### Native status

**Distribution in the natural zone**: Forest steppe and steppe.

##### Distribution

**in Mongolia**: Sel., Tuv. Chogsomzhav (1971):64, Chogsomzhav (1989):91,Sergeev (1995):246, Munkhbat (2010):168, Altanchimeg and Nonnaizab (2013), Altanchimeg et al. (2013b):65, Sergeev et al. (2019):24, Batkhuyag and Batnaran (2021):70.

**Global distribution**: Tuva, SE European Russia, S Siberia, N Kazakhstan, Tien Shan, N Mongolia (Sergeev et al. 2019).

#### Stenobothrus fischeri

(Eversmann, 1848)

82B0519F-E25B-589A-90D3-628A0CBCFEEA

http://orthoptera.speciesfile.org/common/basic/Taxa.aspx/common/Links/Taxa.aspx?TaxonNameID=1107392

##### Native status

**Distribution in the natural zone**: Desert steppe and desert.

##### Distribution

**in Mongolia**: Bulg., Sel., Khovd. Pylnov (1916):277, Günther (1971):116, Chogsomzhav (1972):163, Childebaev and Storozhenko (2001), Sergeev et al. (2009):108, Altanchimeg et al. (2013b):65, Sergeev et al. (2019):23, Batkhuyag and Batnaran (2021):68.

**Global distribution**: Tuva, S Europe, S Siberia (up to Tuva), Asia Minor, Caucasus, Kazakhstan, a mountain of Middle Asia, Mongolia (Sergeev et al. 2019).

#### Stenobothrus lineatus

(Panzer, 1796)

FC81D6D0-0006-5DAD-A98F-982534546747

http://orthoptera.speciesfile.org/common/basic/Taxa.aspx/common/Links/Taxa.aspx?TaxonNameID=1107404

##### Native status

**Distribution in the natural zone**: Forest steppe and steppe.

##### Distribution

**in Mongolia**: Zav., Bulg., Tuv, Khent., S.-baat.,. Cejchan and Maran (1966):180, Steinmann (1967):110, Mistshenko (1968):490, Chogsomzhav (1969b):127, Chogsomzhav (1989):91, Sergeev (1995):246, Childebaev and Storozhenko (2001), Sergeev et al. (2019):23, Batkhuyag and Batnaran (2021):68.

**Global distribution**: Tuva, Europe (except the northern part), S Siberia up to Sakha (Yakutia), Caucasus, N Kazakhstan, N Mongolia, Russian Far East, Dauria (ssp. flavotobialis) (Storozhenko 1985, Sergeev et al. 2019).

#### Megaulacobothrus aethalinus

(Tarbinsky, 1927)

BBBDEE66-7A1D-5B63-8305-F9A64FAD46D3

http://orthoptera.speciesfile.org/Common/basic/Taxa.aspx?TaxonNameID=1106289

##### Native status

**Distribution in the natural zone**: Forest steppe.

##### Distribution

**in Mongolia**: Bogdkhan Mountain, Ulaanbaatar, Altanchimeg et al. (2013a).

**Global distribution**: China (Li et al. 2007), Mongolia (Altanchimeg et al. 2013a).

#### Bryodema gebleri

(Fischer von Waldheim, 1836)

2A2B5F87-1177-5CE1-BA66-D6D62C146A0D

http://orthoptera.speciesfile.org/common/basic/taxa.aspx/common/editimgsnd/Taxa.aspx?TaxonNameID=1104225

##### Native status

**Distribution in the natural zone**: Steppe, desert steppe and desert.

##### Distribution

**in Mongolia**: Uvs. Bey-Bienko (1933):119, Chogsomzhav (1969b):128, Chogsomzhav (1972):180, Sergeev (1995):253, Sergeev et al. (2009):109, Altanchimeg et al. (2015):69, Batkhuyag et al. (2019):107, Sergeev et al. (2020):24, 25, Batkhuyag and Batnaran (2021):101, Dey et al. (2021):335.

**Global distribution**: Tuva, S Ural Mts, Altai-Sayan Mts (except SE Altai), Transbaikalia, Kazakhstan, Tien Shan (except the eastern part), NW China, W Mongolia (Sergeev et al. 2020).

#### Bryodema heptapotanicum

Bey-Bienko, 1930

8EE1E89E-A5D6-5CF2-B1C7-5200FD062686

http://orthoptera.speciesfile.org/common/basic/taxa.aspx/common/editimgsnd/Taxa.aspx?TaxonNameID=1104216

##### Native status

**Distribution in the natural zone**: Desert steppe.

##### Distribution

**in Mongolia**: G.-alt. Altanchimeg and Nonnaizab (2013):81, Batkhuyag and Batnaran (2021):101.

**Global distribution**: China, Xinjiang (Bey-Bienko 1930), Kazakhstan (Childebaev and Storozhenko 2001), Mongolia (Altanchimeg and Nonnaizab 2013).

#### Bryodema kozlovi

Bey-Bienko, 1930

4828320F-B7B1-5A8D-A29C-F74AEBCF7A62

http://orthoptera.speciesfile.org/common/basic/taxa.aspx/common/editimgsnd/Taxa.aspx?TaxonNameID=1104218

##### Native status

**Distribution in the natural zone**: Desert steppe.

##### Distribution

**in Mongolia**: Bey-Bienko (1930):101.

**Global distribution**: Mongolia (Bey-Bienko 1930), China, Inner Mongolia (Alashan) (Zheng et al. 2012).

#### Bryodema luctuosum

(Stoll, 1813)

B37B4047-2432-50E1-B91F-1BC1BDC99196

http://orthoptera.speciesfile.org/common/basic/taxa.aspx/common/editimgsnd/Taxa.aspx?TaxonNameID=1104229

##### Native status

**Distribution in the natural zone**: Steppe, desert steppe and desert.

##### Distribution

**in Mongolia**: Uvs, Zav., Khuvs., A.-khang., Sel., Tuv, Khent., S.-baat., B.-khong., Du.-govi., B.-khong., U-khang. Bolívar (1901):226, 233, Pylnov (1916):279, Bey-Bienko (1930):113, Cejchan and Maran (1966):185, Steinmann (1967):118, Steinmann (1968):247, Mistshenko (1968):495, Chogsomzhav (1968):57, Chogsomzhav (1969b):128, Chogsomzhav (1972):182, Sergeev (1995):254, Altanchimeg and Nonnaizab (2013), Batnaran et al. (2016):37, Batkhuyag and Batnaran (2021):101, Dey et al. (2021):336.

**Global distribution**: Siberia, China and Mongolia (Benediktov 2016).

#### Bryodema miramae

Bey-Bienko, 1930

BC8C9251-4861-5DE6-B9B5-4048587B1635

http://orthoptera.speciesfile.org/common/basic/taxa.aspx/common/editimgsnd/Taxa.aspx?TaxonNameID=1104236

##### Native status

**Distribution in the natural zone**: Desert steppe.

##### Distribution

**in Mongolia**: Altanchimeg and Nonnaizab (2013):81, Batkhuyag and Batnaran (2021):101.

**Global distribution**: Mongolia (Altanchimeg and Nonnaizab 2013), China (Zhang et al. 2006).

#### Bryodema nigripennis

Mistshenko & Gorochov, 1989

5D618B56-9E2F-53ED-82AF-AAFE25ACA24D

http://orthoptera.speciesfile.org/common/basic/taxa.aspx/common/editimgsnd/Taxa.aspx?TaxonNameID=1104220

##### Native status

**Distribution in the natural zone**: Desert steppe.

##### Distribution

**in Mongolia**. B.-khong. Mistshenko and Gorochov (1989):99, Batkhuyag and Batnaran (2021):101.

**Global distribution**: Mongolia (Mistshenko and Gorochov 1989), China (Zhang et al. 2006).

#### Compsorhipis bryodemoides

Bey-Bienko, 1932

F9F6A1B5-CA48-5A4F-854D-55D56962B3AB

http://orthoptera.speciesfile.org/common/basic/taxa.aspx/common/edittaxon/distribution/Taxa.aspx?TaxonNameID=1104250

##### Native status

**Distribution in the natural zone**: Desert steppe and desert.

##### Distribution

**in Mongolia**: Uvs, Bulg., S.-baat., Khovd, G.-alt., B.-khong., U-khang., B.-khong., Du.-govi., U.-govi. Bey-Bienko (1932):84, :606, Cejchan and Maran (1966):186, Mistshenko (1968):495, Chogsomzhav (1968):59, Chogsomzhav (1972):185, Chogsomzhav (1989):94, Sergeev (1995):254, Batnaran et al. (2016):38, Batkhuyag et al. (2019):107, Myagmar et al. (2019):56, Batkhuyag and Batnaran (2021):102, Dey et al. (2021):339.

#### Compsorhipis davidiana

(Saussure, 1888)

0F38FF98-2CB2-57EE-8915-DC32C2517EDD

http://orthoptera.speciesfile.org/common/basic/taxa.aspx/common/edittaxon/distribution/Taxa.aspx?TaxonNameID=1104251

##### Native status

**Distribution in the natural zone**: Desert steppe and desert.

##### Distribution

**in Mongolia**: U.-govi., Uvs, Khovd, B.-khong., Du.-govi. Bolívar (1901):226, 235, Chogsomzhav (1969b):128, Chogsomzhav (1972):184, Chogsomzhav (1989):94, Batkhuyag et al. (2019):107, Sergeev et al. (2020):28, Batkhuyag and Batnaran (2021):102.

**Global distribution**: Tuva, S Transbaikalia, Mongolia, NW, N China (Sergeev et al. 2020).

#### Compsorhipis orientalis

Chogsomzhav, 1989

1AB168F8-3728-5679-97DB-48083529426F

http://orthoptera.speciesfile.org/common/basic/taxa.aspx/common/edittaxon/distribution/Taxa.aspx?TaxonNameID=1104253

##### Native status

**Distribution in the natural zone**: Steppe.

##### Distribution

**in Mongolia**: Do.-govi. Chogsomzhav (1989):94, Batkhuyag and Batnaran (2021):103, Dey et al. (2021):340.

**Global distribution**: China (Yin and Wang 2005), Mongolia (Dey et al. 2021).

#### Bryodemella (Bryodemella) holdereri

(Krauss, 1901)

60C36F3C-092E-5F22-80CF-D5539D3572CF

http://orthoptera.speciesfile.org/common/basic/taxa.aspx/common/specimen/SpecimensByTaxon.aspx?TaxonNameID=1104267

Bryodema occidentale Bey-Bienko (1930):87.

##### Native status

**Distribution in the natural zone**: Forest steppe, steppe, desert steppe and desert.

##### Distribution

**in Mongolia**: Uvs, Zav., Khuvs., A.-khang., Bulg., Sel., Tuv., Khovd, S.-baat., Do., Khovd, G.-alt., B.-khong., U-khang., Du.-govi. Bey-Bienko (1930):85, Chogsomzhav and Shurovenkov (1963):61, Günther (1971):124, Steinmann (1967):119, Steinmann (1968):246, Mistshenko (1968):495, Chogsomzhav (1968):57, Chogsomzhav (1969b):128, Chogsomzhav (1972):179, Sergeev (1995):253, Altanchimeg and Nonnaizab (2005):234, Sergeev et al. (2009):109, Altanchimeg and Nonnaizab (2013), Altanchimeg et al. (2013b):65, Batnaran et al. (2016):37, Sergeev et al. (2020):21, Batkhuyag and Batnaran (2021):99, Dey et al. (2021):337.

**Global distribution**: Tuva, SE Altai, Khakassia, S Krasnoyarsk Region, Transbaikalia, Mongolia, N, NE China (Sergeev et al. 2020).

#### Bryodemella (Bryodemella) tuberculata

(Fabricius, 1775)

88CA8C67-12D4-590B-A28A-B7142A73AE39

http://orthoptera.speciesfile.org/common/basic/taxa.aspx/common/specimen/Taxa.aspx?TaxonNameID=1104270

##### Native status

**Distribution in the natural zone**: Taiga, forest-steppe, steppe and desert steppe.

##### Distribution

**in Mongolia**: B.-Ulg., Uvs, Zav., Khuvs., A.-khang., Bulg., Sel., Tuv, Khent., S.-baat., B.-khong., U-khang., U.-govi. Bolívar (1901):226, Uvarov (1914):171, Pylnov (1916):279, Bey-Bienko (1930):91, Cejchan and Maran (1966):184, Steinmann (1967):118, Steinmann (1968):247, Mistshenko (1968):495, Chogsomzhav (1968):57, Chogsomzhav (1969b):128, Chogsomzhav (1972):179, Batkhuyag and Batnaran (2021):99, Dey et al. (2021):339.

**Global distribution**: Tuva, Europe (except the extreme north and the southern regions of W Europe), Siberia and Far East (northwards to Magadan Region), Kazakhstan, Mongolia, Korea, China, Tibet, Himalayas (Sergeev et al. 2020).

#### Bryodemella (Marikovskiella) orientalis

(Bey-Bienko, 1930)

CDF2D113-5BF7-56DD-9B2F-049B9EA61602

http://orthoptera.speciesfile.org/Common/basic/Taxa.aspx?TaxonNameID=1104280

##### Native status

**Distribution in the natural zone**: Steppe, desert steppe and desert.

##### Distribution

**in Mongolia**: Uvs, Zav., Khovd, G.-alt., B.-khong., U-khang., Du.-govi. Bey-Bienko (1930):101, Bey-Bienko (1933):119, Chogsomzhav (1968):59, Chogsomzhav (1972):180, Sergeev (1995):253, Sergeev et al. (2009):109, Altanchimeg (2011):16, Batnaran et al. (2016):38, Batkhuyag and Batnaran (2021):100.

#### Bryodemella (Marikovskiella) semenovi

(Ikonnikov, 1911)

A818BCB2-1E02-59DF-A969-A189BE26594F

http://orthoptera.speciesfile.org/Common/basic/Taxa.aspx?TaxonNameID=1104279

##### Native status

**Distribution in the natural zone**: High mountain.

##### Distribution

**in Mongolia**: Altanchimeg and Nonnaizab (2013):81, Batkhuyag and Batnaran (2021):100.

**Global distribution**: Kazakhstan (Childebaev and Storozhenko 2001), Mongolia (Altanchimeg and Nonnaizab 2013).

#### Bryodemella (Marikovskiella) zaisanicum fallax

(Bey-Bienko, 1930)

8694B557-1095-561B-9130-75AF140B1C6E

http://orthoptera.speciesfile.org/Common/basic/Taxa.aspx?TaxonNameID=1104285

##### Native status

**Distribution in the natural zone**: Forest steppe.

##### Distribution

**in Mongolia**: B.-Ulg., Uvs, Khovd, Bulg. Günther (1971):125, Bey-Bienko (1930):97, Bey-Bienko (1933):119, Chogsomzhav (1989):95, Sergeev et al. (2009):109, Sergeev et al. (2020):24, Batkhuyag and Batnaran (2021):100.

**Global distribution**: NW Mongolia, NW China, E Kazakhstan (Sergeev et al. 2020).

#### Angaracris barabensis

(Pallas, 1773)

46B16854-4CF1-54DD-9817-5A03072BF416

http://orthoptera.speciesfile.org/Common/basic/Taxa.aspx?TaxonNameID=1104200

Angaracris acrohylina Bi (1986):195.
Angaracris morulimarginis Huang (1981):83.
Angaracris morulipennis Zheng and He. (1994):251.
Angaracris neimongolensis Zheng and Han. (1998):25, 28.
Angaracris nigrimarginis Zheng and Ren. (1993):427.
Angaracris nigripennis Lian Z and Zheng (1984):305.
Oedipoda rhodopa Fischer von Waldheim (1836):348.
Bryodema barabensis var. rhodoptila Karny (1908):49.
Bryodema barabense var. roseipennis Krauss (1901):237.
Angaracris ulashanicus Li (1981):173.
Oedipoda hospes Fischer von Waldheim (1846):295.
Oedipoda lugubris Fischer von Waldheim (1846):298.
Oedipoda thunbergi Stål (1861):345.

##### Native status

**Distribution in the natural zone**: Forest steppe, steppe, desert steppe and desert.

##### Distribution

**in Mongolia**: B.-ulg., Uvs, Zav., Khuvs., A.-khang., Bulg., Tuv., Khent., S.-baat., Do., Khovd, B.-khong., U-khang. Bolívar (1901):226, 223, Pylnov (1916):280, Bey-Bienko (1930):119, Bey-Bienko (1933):119, Chogsomzhav and Shurovenkov (1963), Cejchan and Maran (1966):186, Steinmann (1967):119, Steinmann (1968):267, Mistshenko (1968):495, Chogsomzhav (1968):57-58, Chogsomzhav (1970):128, Chogsomzhav (1972):183, Altanchimeg et al. (2013b):65, Myagmar et al. (2019):56, Sergeev et al. (2020):26-28, Batkhuyag (1995):102, Dey et al. (2021):334.

**Global distribution**: Tuva, S Siberia, Amur Region, N Kazakhstan, Mongolia, N, NE China (Sergeev et al. 2020).

#### Aiolopus thalassinus

(Fabricius, 1781)

CC3F7FA2-C73B-5FD8-BDCC-E1118A82E623

http://orthoptera.speciesfile.org/Common/basic/Taxa.aspx?TaxonNameID=1103315

##### Native status

**Distribution in the natural zone**: Steppe.

##### Distribution

**in Mongolia**: Uvs, A.-khang., Bulg., Khent., S.-baat., Do., B.-khong. Altanchimeg and Nonnaizab (2013):81 which is new registered species in Mongolia.

**Global distribution** S and C Europe, N-Africa, Caucasus, Turkey, Iran, Afghanistan, C Asia, Indian subcontinent, China, SW-Siberia (Garai 2010), South Korea (Storozhenko et al. 2015), Mongolia (Altanchimeg and Nonnaizab 2013).

#### Epacromius pulverulenthus

(Fischer von Waldheim, 1846)

160B394D-387C-5003-946F-763CC6FF81F2

http://orthoptera.speciesfile.org/Common/basic/Taxa.aspx?TaxonNameID=1103429

##### Native status

**Distribution in the natural zone**: Forest steppe, steppe and desert steppe.

##### Distribution

**in Mongolia**: Uvs, A.-khang., Bulg., Khent., S.-baat., Do., B.-khong. Mistshenko (1968):494, Günther (1971):124, Chogsomzhav (1971):86, Chogsomzhav (1972):176, Chogsomzhav (1989):94, Sergeev (1995):251, Childebaev and Storozhenko (2001), Storozhenko et al. (2015):280, Batnaran et al. (2016):38, Myagmar et al. (2019):56, Sergeev et al. (2020):16.

**Global distribution**: South Korea (Storozhenko et al. 2015), Tuva, S Europe, S Siberia, S Russian Far East, Kazakhstan, Tien Shan, Kashmir, Mongolia, China (Sergeev et al. 2020).

#### Epacromius tergestinus

(Megerle von Mühlfeld, 1825)

D5DB0964-AB85-5203-A5FC-76BE144164BF

http://orthoptera.speciesfile.org/Common/basic/Taxa.aspx?TaxonNameID=1103430

Epacromia viridis Uvarov (1910):372.

##### Native status

**Distribution in the natural zone**: Steppe, desert steppe and desert.

##### Distribution

**in Mongolia**: Uvs, Bulg., Sel., Khovd, B.-khong., U.-govi. Pylnov (1916):279, Mistshenko (1968):494, Günther (1971):123, Chogsomzhav (1989):94, Sergeev (1995):251, Childebaev and Storozhenko 2001, Altanchimeg and Nonnaizab (2013), Altanchimeg et al. (2013b):65, Myagmar et al. (2019):56, Sergeev et al. (2020):16, Batkhuyag and Batnaran (2021):95.

**Global distribution**: Tuva, S Europe, S Siberia, Caucasus, Kazakhstan, Tien Shan, Pamiro-Alay, Afghanistan, NW Mongolia, NW China, Tibet (Sergeev et al. 2020).

#### Oedaleus asiaticus

Bey-Bienko, 1941

E057911E-BF1D-5156-B574-2702E31971E9

http://orthoptera.speciesfile.org/common/basic/Taxa.aspx?TaxonNameID=1103223

##### Native status

**Distribution in the natural zone**: Forest steppe, steppe and desert steppe.

##### Distribution

**in Mongolia**: Uvs, Bulg., Sel., Tuv, Khent., S.-baat., Do., Khovd, G.-alt., B.-khong., U-khang., Du.-govi., U.-govi. Bey-Bienko (1941):152, Chogsomzhav and Shurovenkov (1963):61, Chogsomzhav (1968):57, Chogsomzhav (1969b):128, Chogsomzhav (1972):177, Steinmann (1967):118, Steinmann (1968):246, Mistshenko (1968):495, Günther (1971):124, Childebaev and Storozhenko (2001), Altanchimeg and Nonnaizab (2013), Batnaran et al. (2016):38, Myagmar et al. (2019):56, Sergeev et al. (2020):17.

**Global distribution**: Siberia, Kazakhstan, China, Inner Mongolia, Mongolia, Russia (Bey-Bienko 1941, Childebaev and Storozhenko 2001, Sergeev et al. 2020).

#### Oedaleus decorus

(Germar, 1825)

4A0533F8-28D2-5CF9-ACC1-40D001252098

http://orthoptera.speciesfile.org/common/basic/Taxa.aspx?TaxonNameID=1103222

##### Native status

**Distribution in the natural zone**: High mountain.

##### Distribution

**in Mongolia**: Chogsomzhav (1989):93, Sergeev (1995):252, Altanchimeg et al. (2013b):66, Batkhuyag and Batnaran (2021):96, Dey et al. (2021):342.

**Global distribution**: N Africa, Caucasus range, W Asia, C Asia, W Pakistan, Afghanistan, N India (Garai 2010), Mongolia (Dey et al. 2021).

#### Oedaleus infernalis

Saussure, 1884

353B2BC4-3A87-5946-BAD4-98C11422CC50

http://orthoptera.speciesfile.org/common/basic/Taxa.aspx?TaxonNameID=1103218

Oedaleus infernalis montanus Mistshenko and Bey-Bienko (1951):221.
Microgastrimargus taeguensis Lee and Park. (1992):61-64.
Oedaleus infernalis amurensis Ikonnikov, 1911:25

##### Native status

**Distribution in the natural zone**: Steppe and desert steppe.

##### Distribution

**in Mongolia**: G.-alt., Uvs, Sel. Steinmann (1968):246, Pylnov (1916):279, Chogsomzhav (1971):88, Childebaev and Storozhenko (2001), Sergeev et al. (2020):20, Batkhuyag and Batnaran (2021):96.

**Global distribution**: South Korea (Storozhenko et al. 2015), S Russian Far East, NE, E China, Tibet, Japan, Mongolia (Sergeev et al. 2020).

#### Locusta migratoria

(Linnaeus, 1758)

AEFDE635-E4C7-57A6-80C8-49AF8A795067

http://orthoptera.speciesfile.org/Common/basic/Taxa.aspx?TaxonNameID=1103074

##### Native status

**Distribution in the natural zone**: Desert steppe and desert.

##### Distribution

**in Mongolia**: Khovd, B.-khong., U.-govi., Uvs. Chogsomzhav (1968):59, Mistshenko (1968):495, Günther (1971):124, Chogsomzhav (1989):93, Sergeev et al. (2009):109, Altanchimeg and Nonnaizab (2013), Storozhenko et al. (2015):285, Myagmar et al. (2019):56, Sergeev et al. (2020):17, Batkhuyag and Batnaran (2021):96.

**Global distribution**: South Korea (Storozhenko et al. 2015), Tuva, Eurasia (except the north), Africa, Australia and many islands, Mongolia (Sergeev et al. 2020).

#### Psophus stridulus

(Linnaeus, 1758)

6BEB0FAF-D01C-5081-8B73-47AD634B0A76

http://orthoptera.speciesfile.org/Common/basic/Taxa.aspx?TaxonNameID=1103244

Psophus stridulus var. ebneri Karny (1908):57-58.
Nocarodes femoralis Fischer von Waldheim (1846):270.
Acrydium fuliginosum Olivier (1791):223.
Acrydium rubripenne De Geer (1773):472.
Psophus stridulus samniticus Baccetti (1959):397.

##### Native status

**Distribution in the natural zone**: Forest steppe and steppe.

##### Distribution

**in Mongolia**: Du.-govi., Sel. Bolívar (1901):226, Chogsomzhav 1972:178, Chogsomzhav (1989):93, Childebaev and Storozhenko (2001), Altanchimeg and Nonnaizab (2013), Altanchimeg et al. (2013b):65, Batnaran et al. (2016):39, Batkhuyag and Batnaran (2021):98.

**Global distribution**: Mongolia (Altanchimeg et al. 2013b), Tuva, Europe (except the extreme North), S Siberia, S Russian Far East, N Kazakhstan, NE China, Korea (Sergeev et al. 2020).

#### Celes skalozubovi

Adelung, 1906

959E468D-67BE-5B16-A262-793DAB968024

http://orthoptera.speciesfile.org/Common/basic/Taxa.aspx?TaxonNameID=1103549

##### Native status

**Distribution in the natural zone**: Forest steppe, steppe, desert steppe and desert.

##### Distribution

**in Mongolia**: Uvs, Zav., Khuvs., Bulg., Tuv. Bey-Bienko and Mistshenko (1951):587, Mistshenko (1968):495, Chogsomzhav (1969b):128, Chogsomzhav (1972):178, Chogsomzhav (1989):94, Sergeev (1995):252, Altanchimeg and Nonnaizab (2013), Altanchimeg et al. (2013b):65, Batnaran et al. (2016):39, Sergeev et al. (2020):20, Batkhuyag and Batnaran (2021):98, Dey et al. (2021):341.

**Global distribution**: Tuva, S Siberia, N Kazakhstan, Mongolia, N China (Sergeev et al. 2020).

#### Stethophyma grossum

(Linnaeus, 1758)

CB005250-C120-567A-9117-0FB93278E3D7

http://orthoptera.speciesfile.org/Common/basic/Taxa.aspx?TaxonNameID=1104012

Gryllus flavipes Gmelin (1789):2088.
Gryllus (Locusta) germanicus Stoll (1813):41.
Acrydium rubripes De Geer (1773):477.

##### Native status

**Distribution in the natural zone**: Taiga, forest-steppe and steppe.

##### Distribution

**in Mongolia**: B.-Ulg., Uvs, Zav., Khuvs., Tuv, A.-khang., Bulg., Sel., Khent., S.-baat., Khovd, U-khang. Pylnov (1916):279, Bey-Bienko (1933):118, Steinmann (1967):109, Mistshenko (1968):494, Chogsomzhav (1968):57, Chogsomzhav (1969b):127, Chogsomzhav (1972):162, Chogsomzhav (1974b):27, Günther (1971):115, Chogsomzhav (1989):93, Sergeev (1995):251, Childebaev and Storozhenko (2001), Altanchimeg et al. (2013b):65, Storozhenko et al. (2015):272, Batnaran et al. (2016):39, Sergeev et al. (2020):15, Batkhuyag and Batnaran (2021):95.

**Global distribution**: Mongolia, Tuva and almost all temperate Eurasia (except the extreme north) (Sergeev et al. 2020).

#### Sphingoderus carinatus

(Saussure, 1888)

05E05926-2C21-5F49-8AC3-0540F4F48AFF

http://orthoptera.speciesfile.org/Common/basic/Taxa.aspx?TaxonNameID=1103651

##### Native status

**Distribution in the natural zone**: Desert steppe and desert.

##### Distribution

**in Mongolia**: Khovd, U.-govi. Mistshenko (1937):186, Steinmann (1968):247, Chogsomzhav (1968):59, Chogsomzhav (1972):187, Chogsomzhav (1989):95, Childebaev and Storozhenko (2001), Sergeev et al. (2009): 109, Batkhuyag and Batnaran (2021):109.

**Global distribution**: China, Xinjiang, Kazakhstan, Afghanistan, Iran, Mongolia (Childebaev and Storozhenko 2001).

#### Sphingonotus (Sphingonotus) tzaidamicus

Mistshenko, 1937

2BC8276E-E8D4-532D-A910-0FEC78F9D824

http://orthoptera.speciesfile.org/Common/basic/Taxa.aspx?TaxonNameID=1103783

##### Native status

**Distribution in the natural zone**: Desert steppe and desert.

##### Distribution

**in Mongolia**: Do.-govi. Chogsomzhav (1975):45, Chogsomzhav (1989):95, Altanchimeg (2011):16, Myagmar et al. (2019):56, Batkhuyag and Batnaran (2021):104, Dey et al. (2021):350.

**Global distribution**: China (Mistshenko 1937), Mongolia (Dey et al. 2021).

#### Sphingonotus (Sphingonotus) beybienkoi

Mistshenko, 1937

32DF317D-4009-5676-98CC-A9F5CA15AAA5

http://orthoptera.speciesfile.org/Common/basic/Taxa.aspx?TaxonNameID=1103810

##### Native status

**Distribution in the natural zone**: Steppe, desert steppe and desert.

##### Distribution

**in Mongolia**: Uvs, Tuv, G.-alt., B.-khong., U-khang., Khovd, Du.-govi., U.-govi. Mistshenko (1937):148, Mistshenko (1968):495, Günther (1971):128, Chogsomzhav (1972):185, Chogsomzhav (1989):95, Sergeev (1995):255, Sergeev et al. (2009): 109, Altanchimeg et al. (2015):69, Batnaran et al. (2016):39, Myagmar et al. (2019):56, Sergeev et al. (2020):28, Dey et al. (2021):343.

**Global distribution**: Tuva, S Transbaikalia, C, E Kazakhstan, N Kyrgyzstan, Mongolia, NW China (Sergeev et al. 2020).

#### Sphingonotus (Sphingonotus) coerulipes

Uvarov, 1922

3564604C-343E-5918-84E9-3D3E551D227C

http://orthoptera.speciesfile.org/Common/basic/Taxa.aspx?TaxonNameID=1103827

##### Native status

**Distribution in the natural zone**: Desert steppe, taiga and forest-steppe.

##### Distribution

**in Mongolia**: Northwest Mongolia Chogsomzhav (1989):95, Sergeev et al. (2009):109, Popova et al. (2020):604, Batkhuyag and Batnaran (2021):106, Dey et al. (2021):344.

**Global distribution**: Crimea, Lower Volga range, Caucasus, Turkey, Iran, Kazakhstan, Mongolia, S-Siberia (Garai 2010).

#### Sphingonotus (Sphingonotus) elegans

Mistshenko, 1937

C27F7FA9-FB9A-5504-9D5F-F79FB0B83D98

http://orthoptera.speciesfile.org/Common/basic/Taxa.aspx?TaxonNameID=1103719

##### Native status

**Distribution in the natural zone**: Desert steppe and desert.

##### Distribution

**in Mongolia**: Uvs, Khovd, B.-khong., U.-govi., U-khang. Mistshenko (1937):165, Mistshenko (1968):495, Chogsomzhav (1968):59, Chogsomzhav (1972):185, Günther (1971):128, Chogsomzhav (1989):95, Garai (2001):752, Sergeev et al. (2009):109, Batnaran et al. (2016):40, Myagmar et al. (2019):56, Sergeev et al. (2020):29, Dey et al. (2021):343.

**Global distribution**: The Mongolian part of Uvs-Nuur Intermountain Basin (Chogsomzhav 1974b), Middle Asia, NW China (Sergeev et al. 2020).

#### Sphingonotus (Sphingonotus) gobicus

Chogsomzhav, 1975

E053D09E-57CA-59AE-BCDB-33D4A1E7A785

http://orthoptera.speciesfile.org/Common/basic/Taxa.aspx?TaxonNameID=1103705

##### Native status

**Distribution in the natural zone**: Desert steppe and desert.

##### Distribution

**in Mongolia**: Khovd, G.-alt., B.-khong., Do.-govi, Chogsomzhav (1975):44, Chogsomzhav (1989):95, Batkhuyag and Batnaran (2021):105, Dey et al. (2021):345.

#### Sphingonotus (Sphingonotus) salinus

(Pallas, 1773)

E22042BD-4529-5729-BBA7-F1B2FEEC5DD4

http://orthoptera.speciesfile.org/Common/basic/Taxa.aspx?TaxonNameID=1103776

Sphingonotus suschkini Adelung (1906):86.
Oedipoda zinini Kittary (1849):470.

##### Native status

**Distribution in the natural zone**: Desert steppe and desert.

##### Distribution

**in Mongolia**: Khovd. Chogsomzhav (1969a):78, Chogsomzhav (1972):186, Chogsomzhav (1989):95, Sergeev (1995):255, Sergeev et al. (2009):109, Myagmar et al. (2019):56, Sergeev et al. (2020):29, Batkhuyag and Batnaran (2021):108.

**Global distribution**: Tuva, SE Europe, Caucasus (deserts), Kazakhstan (semi-deserts and deserts), Middle Asia, NW China, NW Mongolia (Sergeev et al. 2020).

#### Sphingonotus (Sphingonotus) halophilus

Bey-Bienko, 1929

0926D801-CFDC-53D0-8A34-3DCE90C9ECAD

http://orthoptera.speciesfile.org/Common/basic/Taxa.aspx?TaxonNameID=1103732

##### Native status

**Distribution in the natural zone**: Desert and desert steppe.

##### Distribution

**in Mongolia**: Khovd. Günther (1971):128, Batkhuyag and Batnaran (2021):104.

**Global distribution**: Mongolia (Günther 1971), SE part of European Russia, Kazakhstan (Sergeev 2021).

#### Sphingonotus (Sphingonotus) mongolicus

Saussure, 1888

9EEB0DE8-4AA4-5D1C-B57A-614F2DE63B3A

http://orthoptera.speciesfile.org/Common/basic/Taxa.aspx?TaxonNameID=1103759

##### Native status

**Distribution in the natural zone**: Taiga, forest-steppe and steppe.

##### Distribution

**in Mongolia**: Tuv, B.-khong., U.-govi. Saussure (1888):77, 82, Mistshenko (1937):229, Mistshenko (1968):496, Chogsomzhav (1971):106, Chogsomzhav (1972):186, Chogsomzhav (1989):95, Nonnaizab et al. (1999):15, Altanchimeg and Nonnaizab 2013, Altanchimeg et al. (2013b):65, Batkhuyag and Batnaran (2021):108, Dey et al. (2021):346.

#### Sphingonotus (Sphingonotus) nebulosus

(Fischer von Waldheim, 1846)

815495B1-5CF8-5F8A-9C5E-E5E90B5EBF72

http://orthoptera.speciesfile.org/Common/basic/Taxa.aspx?TaxonNameID=1103863

##### Native status

**Distribution in the natural zone**: Desert.

##### Distribution

**in Mongolia**: Khovd, U.-govi. Chogsomzhav (1968):58, Chogsomzhav (1971):106, Chogsomzhav (1972):186, Chogsomzhav (1975):44, Chogsomzhav (1989):95, Günther (1971):128, Sergeev (1995):255, Myagmar et al. (2019):56, Sergeev et al. (2020):29, Batkhuyag and Batnaran (2021):107.

**Global distribution**: Tuva, SE Altai, Asia Minor, Caucasus, Kazakhstan (except the north), Tien Shanzubo, Pamiro-Alay, Iran, Pakistan, NW Mongolia, NW China (Sergeev et al. 2020).

#### Sphingonotus (Sphingonotus) obscuratus latissimus

Uvarov, 1925

BD1C19A3-D989-5CDA-BBC3-E513DD11C9F7

http://orthoptera.speciesfile.org/Common/basic/Taxa.aspx?TaxonNameID=1103875

##### Native status

**Distribution in the natural zone**: Desert steppe and desert.

##### Distribution

**in Mongolia**: Khovd, B.-khong., U.-govi. Mistshenko (1968):491, Chogsomzhav (1968):59, Chogsomzhav (1971):107, Chogsomzhav (1972):186, Günther (1971):129, Chogsomzhav (1989):95, Childebaev and Storozhenko (2001), Batkhuyag and Batnaran (2021):108, Dey et al. (2021):348.

**Global distribution**: Kazakhstan and Mongolia (Childebaev and Storozhenko 2001).

#### Sphingonotus (Sphingonotus) rubescens

(Walker, 1870)

4E506E93-B94B-5E01-81C7-678F0E42BA51

http://orthoptera.speciesfile.org/Common/basic/Taxa.aspx?TaxonNameID=1103882

##### Native status

**Distribution in the natural zone**: High mountain and desert steppe.

##### Distribution

**in Mongolia**: Uvs, U.-govi. Chogsomzhav (1969a):77, Chogsomzhav (1969b):128, Chogsomzhav (1972):185, Chogsomzhav (1989):95, Sergeev (1995):255, Sergeev et al. (2009):109, Batnaran et al. (2016):40, Myagmar et al. (2019):56, Sergeev et al. (2020):29, Batkhuyag and Batnaran (2021):105, Dey et al. (2021):348.

**Global distribution**: The Mongolian part of Uvs-Nuur Intermountain Basin (Chogsomzhav 1974b). Arid part of N Caucasus, N Africa, SW Asia, deserts of Middle Asia (including mountains), NW Mongolia, NW China (Sergeev et al. 2020).

#### Helioscirtus moseri

Saussure, 1884

F067BC7E-EA2E-56F1-A4E3-97133897B45B

http://orthoptera.speciesfile.org/Common/basic/Taxa.aspx?TaxonNameID=1103593

##### Native status

**Distribution in the natural zone**: Desert.

##### Distribution

**in Mongolia**: G.-alt. Chogsomzhav (1969b), Batkhuyag and Batnaran (2021):109.

**Global distribution**: Mongolia (Chogsomzhav 1969b), northern Africa and western Asia (Hodjat et al. 2018).

#### Leptopternis gracilis

(Eversmann, 1848)

B1C152B3-4417-50D3-ACFD-F734819C9CE9

http://orthoptera.speciesfile.org/Common/basic/Taxa.aspx?TaxonNameID=1104641

Hyalorrhipis maculipennis Saussure (1884):195.
Sphingonotus angustipennis Werner (1905):201.
Sphingonotus grobbeni Chopard (1949):361.

##### Native status

**Distribution in the natural zone**: Desert steppe and desert.

##### Distribution

**in Mongolia**: U.-govi. Mistshenko (1968):496, Chogsomzhav (1972):187, Chogsomzhav (1989):95, Garai (2001):753, Childebaev and Storozhenko (2001), Batkhuyag et al. (2019):107, Myagmar et al. (2019):57, Batkhuyag and Batnaran (2021):109.

**Global distribution**: N Africa, Lower Volga range, Caucasus range, Iran, Afghanistan, C Asia, W China, Mongolia (Garai 2001).

#### Leptopternis iliensis

Uvarov, 1925

2941A25C-888F-5524-9E9E-08D9DCA69A37

http://orthoptera.speciesfile.org/Common/basic/Taxa.aspx?TaxonNameID=1104645

##### Native status

**Distribution in the natural zone**: Desert steppe.

##### Distribution

**in Mongolia**: Khovd. Günther (1971):129, Chogsomzhav (1989):95, Childebaev and Storozhenko (2001), Batkhuyag and Batnaran (2021):109.

**Global distribution**: Kazakhstan and Mongolia (Childebaev and Storozhenko 2001).

#### Bohemanella frigida

(Boheman, 1846)

3E348E31-A2B9-5CA1-9CE3-5F284E300D43

http://orthoptera.speciesfile.org/Common/basic/Taxa.aspx?TaxonNameID=1110374

##### Native status

**Distribution in the natural zone**: Taiga and forest-steppe.

##### Distribution

**in Mongolia**: Uvs, Zav., Khuvs., Tuv. Uvarov (1914):17, Mistshenko (1952):425, Mistshenko (1968):489, Steinmann (1968):240, Cejchan and Maran (1966):178, Chogsomzhav (1969b):127, Chogsomzhav (1972):154, Chogsomzhav (1989):90, Günther (1971):113, Munkhbat (2010):169, Altanchimeg et al. (2013b):64, Sergeev et al. (2019):14, Batkhuyag and Batnaran (2021):44.

**Global distribution**: Tuva, N Eurasia (in the southern parts of Europe in mountains), Alaska, N Canada, Mongolia (Sergeev et al. 2019).

#### Podisma pedestris

(Linnaeus, 1758)

C690954A-FA32-5582-B234-C967CBB37ACD

http://orthoptera.speciesfile.org/Common/Basic/Taxa.aspx?TaxonNameID=1111031

##### Native status

**Distribution in the natural zone**: Taiga and forest-steppe.

##### Distribution

**in Mongolia**: Sel., Khuvs. Mistshenko (1952), Chogsomzhav (1972):154, Chogsomzhav (1989):90, Altanchimeg et al. (2013b):64, Sergeev et al. (2019):13, Batkhuyag and Batnaran (2021):43.

**Global distribution**: Tuva, Europe, W Siberia (forest steppes and steppes), E Siberia (up to the central parts of Yakutia), mountains of S Siberia, N Caucasus, NW and N Kazakhstan, Dzungarian Alatau, E Tien Shan, Tarbagatai Mts, N Mongolia (Sergeev et al. 2019).

#### Prumna primnoa

(Motschulsky, 1846)

BB2A8612-82B9-5924-82AF-F38F39CC4A26

http://orthoptera.speciesfile.org/Common/basic/Taxa.aspx?TaxonNameID=1111290

Podisma sachalinensis Matsumura (1911):5.
Podisma viridis Fischer von Waldheim (1846):248.

##### Native status

**Distribution in the natural zone**: Forest steppe and steppe.

##### Distribution

**in Mongolia**: Khent., Sel., Tuv. Pylnov (1916):280, Cejchan and Maran (1966): 178, Chogsomzhav (1989):90, Batnaran (2008):43, Batnaran et al. (2016):36, Sergeev et al. (2019):13, Batkhuyag and Batnaran (2021):43.

**Global distribution**: Tuva, S Siberia, the southern part of Russian Far East (except the southern part of Primorsky Region), including Sakhalin and Kunashir, N Mongolia (Sergeev et al. 2019).

#### Ognevia longipennis

(Shiraki, 1910)

E435C8A7-497B-559C-BC3E-5E72990CC630

http://orthoptera.speciesfile.org/Common/Basic/Taxa.aspx?TaxonNameID=1111098

Eirenephilus debilis Ikonnikov (1911):265.
Podisma alpina niphona Furukawa (1929):171,177.

##### Native status

**Distribution in the natural zone**: Taiga and forest steppe.

##### Distribution

**in Mongolia**: Khent., Sel. Bey-Bienko and Mistshenko (1951):236, Chogsomzhav (1989):90, Storozhenko and Paik (2007):157, Altanchimeg et al. (2013b):64, Storozhenko et al. (2015):220, Sergeev et al. (2019):13, Batkhuyag and Batnaran (2021):45.

**Global distribution**: Tuva, S Siberia (mainly in mountains), S Russian Far East, including Sakhalin and S Kurile Islands, E Kazakhstan, N Mongolia, N, NE China, Korea, Japan (Sergeev et al. 2019).

#### Zubovskya koeppeni

(Zubovski, 1900)

67E5FBE4-8AD6-5CF3-ADF4-D38B1A35E1A9

http://orthoptera.speciesfile.org/Common/basic/Taxa.aspx?TaxonNameID=1111120

##### Native status

**Distribution in the natural zone**: Forest steppe.

##### Distribution

**in Mongolia**: Khuvs. Chogsomzhav (1989):90, Batkhuyag and Batnaran (2021):43.

**Global distribution**: Mongolia (Chogsomzhav 1989) , Tuva, WSE, Altay-Sayan Mts. including W Altay (Sergeev et al. 2019).

#### Zubovskya mongolica

Storozhenko, 1986

AD25690D-C195-5001-8D98-F2F13B28318A

http://orthoptera.speciesfile.org/Common/basic/Taxa.aspx?TaxonNameID=1111118

##### Native status

**Distribution in the natural zone**: Forest steppe.

##### Distribution

**in Mongolia**: Khuvs. Storozhenko (1986):53, Sergeev et al. (2019):12.

**Global distribution**: Mongolia and Siberia (Storozhenko 1986, Sergeev et al. 2019).

#### Calliptamus abbreviatus

Ikonnikov, 1913

7E98110A-2BB1-5B24-A44D-0280C8AF4321

http://orthoptera.speciesfile.org/Common/basic/Taxa.aspx?TaxonNameID=1112890

Calliptamus doii Lee and Lee (1985):24.
Calliptamus abbreviatus f. holoptera Ramme (1952):308.
Calliptamus sibiricus Wnukowskij (1926):91.

##### Native status

**Distribution in the natural zone**: High mountain, forest steppe, desert steppe and desert.

##### Distribution

**in Mongolia**: Uvs, Zav., Khuvs., Bulg., Sel., Khent., Do., Khovd, B.-khong, U-khang, Du.-govi., U.-govi. Pylnov (1916):280, Mistshenko (1952):531, Mistshenko (1968):489, Steinmann (1968):240, Chogsomzhav (1968):57, Chogsomzhav (1969b):127, Chogsomzhav (1972):155, Chogsomzhav (1975):38, Günther (1971):113, Batnaran (2008):44, Sergeev (1995):240, Childebaev and Storozhenko (2001):25, Altanchimeg (2011):16, Altanchimeg and Nonnaizab 2013, Altanchimeg et al. (2013b):65, Storozhenko et al. (2015):225, Batnaran et al. (2016):31, Sergeev et al. (2019):15, Myagmar et al. (2019):56, Batkhuyag and Batnaran (2021):45.

**Global distribution**: Tuva, S Siberia (from the south-eastern part of W Siberian Plain to Daura), the southern part of the Russian Far East, NE, E Kazakhstan, N Mongolia, N, NE, E China, South Korea (Sergeev et al. 2019).

#### Calliptamus barbarus cephalotes

Fischer-Waldheim, 1846

78ADBACC-59FE-5EEA-8355-51D8B93BDE02

http://orthoptera.speciesfile.org/Common/basic/Taxa.aspx?TaxonNameID=1112930

##### Native status

**Distribution in the natural zone**: Steppe, desert steppe and desert.

##### Distribution

**in Mongolia**: Khovd, G.-alt., B.-khong., U.-govi. Mistshenko (1952):544, Mistshenko (1968):489, Chogsomzhav (1968):59, Chogsomzhav (1989):90, Childebaev and Storozhenko (2001):25, Garai (2001):402, Altanchimeg and Nonnaizab (2013), Batnaran et al. (2016):31, Myagmar et al. (2019):56, Batkhuyag and Batnaran (2021):46.

**Global distribution**: N Africa, Caucasus, Turkey, Iran, N-Afghanistan, Kazakhstan, Mongolia, W China, Siberia (Garai 2001).

#### Calliptamus italicus

(Linnaeus, 1758)

D80D32C7-F63E-561C-B3AD-AB814853C1CA

http://orthoptera.speciesfile.org/Common/basic/Taxa.aspx?TaxonNameID=1112938

##### Native status

**Distribution in the natural zone**: Steppe.

##### Distribution

**in Mongolia**: Khuvs. Mistshenko and Bey-Bienko (1951):256, Chogsomzhav (1971):53, Chogsomzhav (1989):90, Childebaev and Storozhenko (2001):25, Sergeev et al. (2009):108, Altanchimeg and Nonnaizab 2013, Batkhuyag and Batnaran (2021):46.

**Global distribution**: N Africa, Turkey, from the Caucasus through Iran, Afghanistan, W Pakistan, to NW Mongolia, W China and W Siberia (Garai 2001).

#### Dericorys annulata

(Fieber, 1853)

B9FF2057-1574-50DF-B38B-E81F4961605D

http://orthoptera.speciesfile.org/Common/basic/Taxa.aspx?TaxonNameID=1117733

Dericorys lazurescens Uvarov (1914):142, 146.
Derocorystes (Cyphophorus) roseipennis Redtenbacher (1889):30.

##### Native status

**Distribution in the natural zone**: Desert steppe.

##### Distribution

**in Mongolia**: Khovd, B.-khong., U.-govi. Mistshenko and Bey-Bienko (1951):151, Mistshenko (1952):97, Mistshenko (1968):489, Chogsomzhav (1968):59, Chogsomzhav (1972):153, Chogsomzhav (1974b):26, Steinmann (1971):146, Childebaev and Storozhenko (2001):21, Altanchimeg et al. (2015):69, Myagmar et al. (2019):57, Batkhuyag and Batnaran (2021):39.

**Global distribution**: Kazakhstan, Afghanistan, China, Mongolia (Childebaev and Storozhenko 2001).

#### Beybienkia lithophila

Gorochov & Mistshenko, 1989

CB63B169-7B18-561B-9FAD-D6C812DE8CF6

http://orthoptera.speciesfile.org/Common/basic/Taxa.aspx?TaxonNameID=1117444

##### Native status

**Distribution in the natural zone**: Desert steppe.

##### Distribution

**in Mongolia**: B.-khong. Podgornaya and Gorochov 1989:105, Batkhuyag and Batnaran 2021:37.

**Global distribution**: Mongolia (Ünal 2016).

#### Beybienkia songorica

Tsyplenkov, 1956

90248C8C-5C5A-5B8D-B551-8FA594334504

http://orthoptera.speciesfile.org/Common/basic/Taxa.aspx?TaxonNameID=1117445

##### Native status

**Distribution in the natural zone**: Desert.

##### Distribution

**in Mongolia**: Altai, Transaltai gobi Chogsomzhav (1989):89, Batkhuyag and Batnaran (2021):37.

**Global distribution**: Mongolia (Chogsomzhav 1989), China (Tsyplenkov 1956).

#### Mongolotmethis gobiensis

Bey-Bienko, 1948

7AABA4C9-C9DB-5CB4-8BDC-30F6C998797E

http://orthoptera.speciesfile.org/Common/basic/Taxa.aspx?TaxonNameID=1117481

##### Native status

**Distribution in the natural zone**: Steppe and desert steppe.

##### Distribution

**in Mongolia**: B.-khong., U.-govi.,U-khang. Bey-Bienko (1948):9, Bey-Bienko and Mistshenko (1951):321, Mistshenko (1968):489, Chogsomzhav (1968):58, Altanchimeg (2011):16, Batkhuyag et al. (2014):78, Myagmar et al. (2019):57, Batkhuyag and Batnaran (2021):34.

**Global distribution**: China, Inner Mongolia (Alashan), Mongolia (Batkhuyag et al. 2014).

#### Mongolotmethis kozlovi

Bey-Bienko, 1948

8113C81C-ABF9-5096-AF87-C8D56F7AC212

http://orthoptera.speciesfile.org/Common/basic/Taxa.aspx?TaxonNameID=1117480

##### Native status

**Distribution in the natural zone**: Steppe and desert steppe

##### Distribution

**in Mongolia**: Tuv, Du.-govi., B.-khong., U.-govi. Bey-Bienko (1948):10, Bey-Bienko and Mistshenko (1951):321, Mistshenko (1968):489, Chogsomzhav (1968):58, Altanchimeg (2011):16, Batkhuyag et al. (2014):78, Myagmar et al. (2019):57, Batkhuyag et al. (2019):107, Batkhuyag and Batnaran (2021):34.

#### Mongolotmethis michidi

Batkhuyag, Batnaran & Dorjderem, 2014

37B91251-248A-5759-89AD-8DBB1541C6C0

http://orthoptera.speciesfile.org/Common/basic/Taxa.aspx?TaxonNameID=1221002

##### Native status

**Distribution in the natural zone**: Steppe and desert steppe.

##### Distribution

**in Mongolia**: G.-alt., U.-govi. Batkhuyag et al. (2014), Batkhuyag and Batnaran (2021):35.

#### Rhinotmethis beybienkoi

Chogsomzhav, 1975

DF52ED2C-2F71-5FB8-91EB-C350199E46C7

http://orthoptera.speciesfile.org/Common/basic/Taxa.aspx?TaxonNameID=1117505

##### Native status

**Distribution in the natural zone**: Desert steppe and desert.

##### Distribution

**in Mongolia**: Du.-govi. Chogsomzhav (1975):39, Gorochov et al. (1989), Chogsomzhav (1989):89, Altanchimeg (2011):16, Batkhuyag et al. (2014):78, Batnaran et al. (2016):30, Ünal (2016):59, Batkhuyag and Batnaran (2021):36.

**Global distribution**: Mongolia (Ünal 2016).

#### Rhinotmethis hummeli

Sjöstedt, 1933

D06115E5-16B2-5A6A-99AB-3149A477F098

http://orthoptera.speciesfile.org/Common/basic/Taxa.aspx?TaxonNameID=1117506

##### Native status

**Distribution in the natural zone**: Desert steppe and desert.

##### Distribution

**in Mongolia**: Do.-govi. Bey-Bienko (1948):12, Chogsomzhav (1975):33, Chogsomzhav (1989):89, Sergeev (1995):233, Ünal (2016):59, Myagmar et al. (2019):57.

**Global distribution**: China, Inner Mongolia, Mongolia (Ünal 2016, Batkhuyag and Batnaran 2021).

#### Asiotmethis similis

Bey Bienko, 1951

24D1A28B-2443-57D5-8375-23D25DAC92C5

http://orthoptera.speciesfile.org/Common/basic/Taxa.aspx?TaxonNameID=1117408

##### Native status

**Distribution in the natural zone**: Forest steppe and desert steppe.

##### Distribution

**in Mongolia**: G.-alt. Chogsomzhav (1989):89, Batkhuyag and Batnaran (2021):31.

**Global distribution**: Central Asia, Kazakhstan (Bey-Bienko and Mistshenko 1951), Mongolia (Chogsomzhav 1989).

#### Haplotropis brunneriana

Saussure, 1888

79A6A339-1957-5559-8897-1494563414D4

http://orthoptera.speciesfile.org/Common/basic/Taxa.aspx?TaxonNameID=1116957

Sulcotropis cyanipes Yin and Chou. (1979):128.
Staurotylus mandshuricus Adelung (1910):344.
Haplotropis neimongolensis Jin (1994):251

##### Native status

**Distribution in the natural zone**: Forest steppe and steppe.

##### Distribution

**in Mongolia**: Do. Chogsomzhav (1975):41, Chogsomzhav (1989):95, Storozhenko and Paik (2007):133, Storozhenko et al. (2015):184, Batnaran et al. (2016):31, Ünal (2016):60, Batkhuyag and Batnaran (2021):38.

**Global distribution**: China, Inner Mongolia (Jin 1994), Manchuria (Yin and Chou. 1979), Russian Far East, eastern Asia, South Korea, China North-central, Mongolia (Storozhenko and Paik 2007, Ünal 2016).

## Analysis

### Result

The present study aimed to list the rare and unexplored species of grasshoppers (except long-horned grasshopper and cricket species) in Mongolia. Currently, the grasshopper fauna of Mongolia comprises 128 species, which are distributed in 52 genera and 19 tribes (Table 2). Of these, 34 species are also included in the Checklist of European Orthoptera (Acridoidea) (Suppl. material 2). In addition, Mongolia's distribution of grasshoppers was divided into six distinct natural zones (Fig. 3), (Suppl. material 3). Additionally, a combined cluster analysis was performed using the grasshopper component numbers between neighbouring countries, including Russia, China and South Korea (Suppl. material 4).

**Figure 1.**
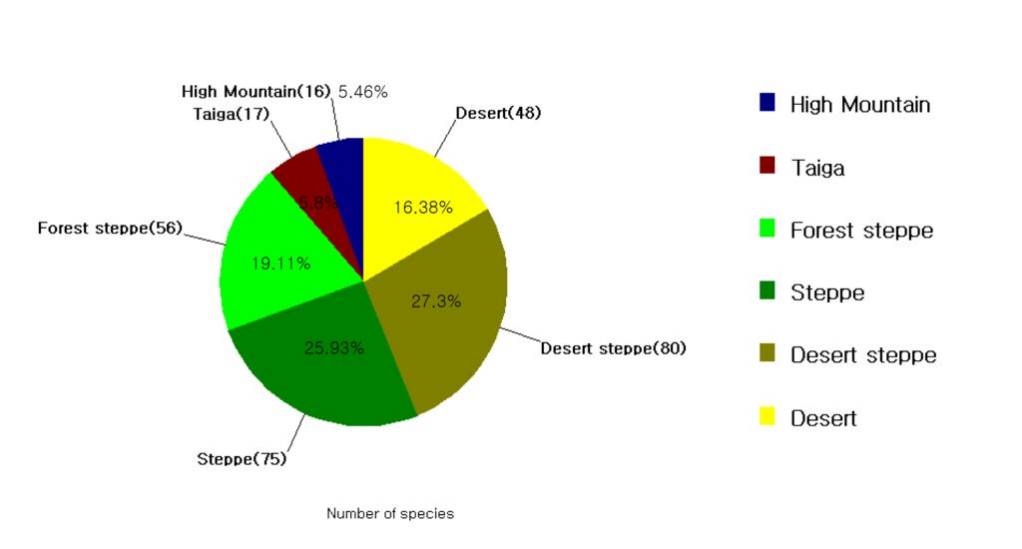
The grasshopper distribution of six different natural zones in Mongolia. Desert steppe - 80 species (27.30%), Steppe - 75 species (25.93%), Forest steppe - 56 species (19.11%), Desert - 48 species (16.38%), Taiga - 17 species (5.80%), High Mountain - 16 species (5.46%) distributed, respectively.

**Figure 2.**
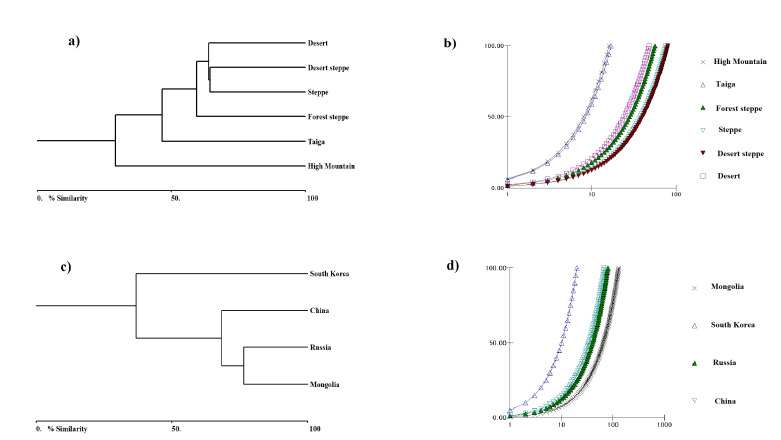
Dendrogram showing the Bray-Curtis cluster analysis (Single link) of distribution of grasshopper species in six different natural zones (**a**); Species distribution rank abundance plot of Mongolian grasshopper by Natural Zone (**b**); Bray-Curtis cluster analysis (single link) neighbouring boundary countries (Russia, China and South Korea) grasshopper distribution (**c**); Abundance Plot of boundary countries (Russia, China including South Korea) grasshopper distribution (**d**).

**Figure 3.**
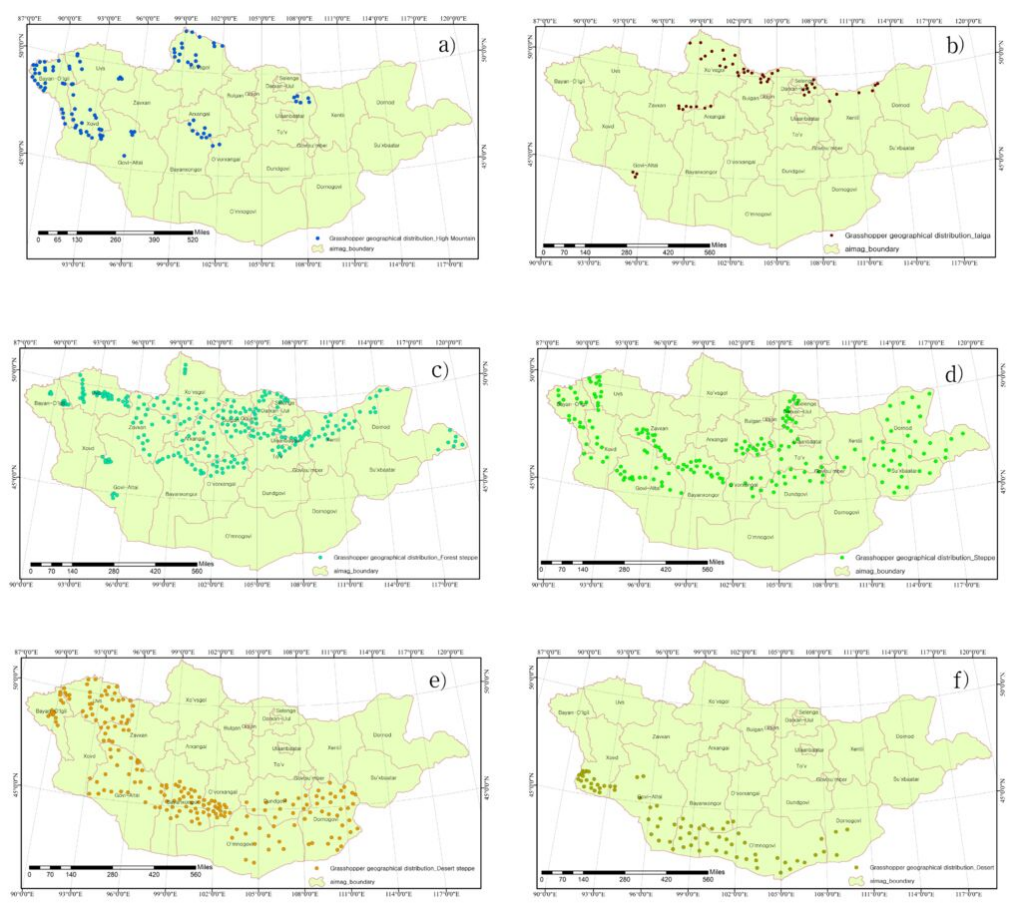
Distribution of grasshoppers in Mongolian natural zones in the High mountain natural zone (**a**); taiga natural zone (**b**); forest steppe natural zone (**c**); steppe natural zone (**d**); desert steppe natural zone (**e**); and desert natural zone (**f**).

The rich distribution of Mongolian grasshoppers was characterised by six habitats using the remnant natural habitat and forest types (Fig. 1). The total number of grasshopper species in each natural zone was as follows: 75 species (25.93%) in the steppe, 16 species (5.46%) in the high mountains, 56 species (19.11%) in the forest-steppe, 80 species (27.30%) in the desert steppe, 17 species (5.80%) in the taiga and 48 species (16.38%) in the desert. The similarity matrix between the geographical distribution of grasshopper results presented a high mountain and taiga zone of 12.1%, taiga and forest-steppe zone of 46.57%, forest-steppe and steppe zone of 59.54%, steppe and desert steppe zone of 64.51% and desert steppe and desert zone of 64.06% (Table 1). The species natural zone results used by a single-link Bray-Curtis cluster analysis dendrogram (Fig. 2a, b) considered desert and desert steppe zones, steppe and forest-steppe zones and high mountain and taiga zones as most closely related. The high mountain and taiga zones were less distributed than the other zones. In addition, the Shannon index and Berger–Parker's index showed six different natural zones (Suppl. material 3). Desert steppe and steppe zones were the most distributed. In contrast, the high mountain and taiga zones were less distributed. Grasshoppers are widely distributed in the desert steppe and steppe natural zone (Suppl. material 1). Twenty grasshopper species that are extensively dispersed in the desert-steppe natural zone have been identified as the indicator species of this zone. These species are listed as follows: *Rhinotmethishummeli* Sjost.* *Dericorysannulata* (Fieb.), *Calliptamusbarbaruscephalotes* F.-W., *Acridakozlovi* Mistsh., *Arcypterameridionalis* Ikonn., *Arcypteramicroptera* (F.-W.), *Stenobothrusfischeri* Ev., *Celesskalozubovi* Adel., *Compsorhipisbryodemoides* B.-Bien.*, *Leptopternisgracilis* (Ev.), *Sphingoderuscarinatus* (Sauss.) *Sphingonotusbeybienkoi* Mistsh., *Sphingonotuscoerulipes* Uv., *Sphingonotuselegans* Mistsh., *Sphingonotusgobicus* Chogs.*, *Sphingonotusnebulosus* (F.-W.), *Sphingonotusobscuratuslatissimus* Uv., *Sphingonotusrubescens* (Walker), *Sphingonotussalinus* (Pall.) and *Sphingonotustzaidamicus* Mistsh. The indicator species of each of the six types of natural zones are identified by (★) abbreviations (Suppl. material 1). In total, 16 species (13.17%) were endemic and were commonly distributed in desert steppe and desert natural zones. The similarity matrix showed the species distribution of grasshoppers in Mongolia, Russia, China and South Korea. A single-link Bray-Curtis cluster analysis dendrogram was constructed using the combined distribution data for all species. The results exhibited the relationship between a Mongolian grasshopper species and species in Russia and China that were most closely related to the Mongolian species. South Korea was reported as the country that was most distantly linked to Mongolia (Fig. 2). The number of Mongolian grasshopper species was compared with those of grasshopper species in the neighbouring countries, such as Russia and China and South Korea was included using a single-link Bray-Curtis cluster analysis dendrogram (Fig. 2c). The similarity index between the number of species of Mongolian grasshopper and those of grasshopper species in China, Russia and South Korea was 68.36%, 76.55% and 26.84%, respectively. Furthermore, grasshopper species from China showed 36.78% and 65.30% similarity with those in South Korea and Russia, respectively. South Korea showed that 36% of the Russian-distributed species were similar (Table 3). In summary, Russia and China are closely tied to the number of Mongolian grasshopper species, whereas South Korea is distantly related.

In addition, 17 species of grasshoppers are endemic to Mongolia (13.17%), including *Mongolotmethisgobiensis* B.-Bien, *Mongolotmethiskozlovi* B.-Bien, *Rhinotmethishummeli* Sjost, *Podismopsisaltaica* Zub, *Eclipophlepsbogdanovi* Tarb, *Eclipophlepscarinata* Mistsh, *Eclipophlepsconfinis* Mistsh, *Eclipophlepsglacialis* B.-Bien, *Eclipophlepskerzhneri* Mistsh, *Eclipophlepslucida* Mistsh, *Eclipophlepssimilis* Mistsh, *Eclipophlepstarbinskii* Oristsh, *Stenobothrusnewskii* Zub, *Bryodemagebleri* (F.-W.), Bryodema (M.) orientalis B.-Bien and *Compsorhipisbryodemoides* B.-Bien. Also, Chorthippus (G.) mollis (Charp.), Chorthippus (G.) vagans (Ev.), Chorthippus (M.) chinensis Tarb., *Aiolopusthalassinus* (Fabr.), *Bryodemaheptapotanicum* B.-Bien., *Bryodemamiramae* B.-Bien, Bryodema (M.) semenovi Ikonn., *Sphingonotusgobicus* Chogs. species are new identified species from Mongolia Altanchimeg and Nonnaizab (2013) (Suppl. material 1). Furthermore, 34 grasshopper species are registered on the European Red List and two of them are listed as Endangered, four are listed as Near Threatened and 28 are classified as Least Concern (Suppl. material 2).

## Discussion

The grasshopper fauna of Mongolia comprises 128 species, including three families, eight subfamilies, 19 tribes and 52 genera, of which 34 species are registered on the European Red List (Table 2). The 17 grasshopper species that are considered endemic to Mongolia are distributed in desert and desert steppe. Notably, some species are widely distributed in neighbouring countries, such as Russia, China and South Korea. The taxonomic keys of the superfamily Acridoidea in Mongolia were recently updated and it was reported that the superfamily includes three families, 49 genera and 127 species (Batkhuyag and Batnaran 2021). A difference of six species was observed between our checklist and the list updated by Batkhuyag and Batnaran (2021). These species included *Gomphoceruslicenti* Chang, Chorthippus (Ch.) karelini Uv., *Stenobothruskirgizorum* Ikonn., *Sphingonotushalocnemi* Uv., *Sphingonotusturcmenus* B.-Bien. and *Eclipophlepsconfinislevis* Mistsh. In addition, the records of specimens of four species were uncertain; these included Chorthippus (G.) buyanticus Batkhuyag et al., Chorthippus (G.) tseelicus Batlhuyag et al., *Sphingonotushalocnemi* Uv. and *Sphingonotusturcmenus* B.-Bien. In the present study, four species with uncertain distribution sources, namely *Gomphoceruslicenti* Chang, Chorthippus (Ch.) karelini Uv., *Stenobothruskirgizorum* Ikonn. and *Eclipophlepslevis* Mistsh, were excluded from our checklist. In the future, the species list should be updated after previously unreported species have been identified and reported by undertaking additional research.

We compared the findings of our checklist with those of Batkhuyag and Batnaran (2021) and we found discrepancies related to three genera, namely *Aiolopus* Fieber, 1853, *Pseudochorthippus* Defaut, 2012 and *Megaulacobothrus* Caudell, 1921, as well as three species, which includes *Aeropedellusbaliolus* Mistsh., *Bryodemakozlovi* B.-Bien., and *Aiolopusthalassinus* (Fabr.). These genus species are widely distributed in forests and steppe in Mongolia. When creating an annotated checklist, the species that were identified from literature were divided into natural zones, based on their distribution. However, Mongolia has the world’s largest intact grassland with respect to its biodiversity (Batsaikhan et al. 2014, Herbert et al. 2019), which has great importance for the preservation of native vascular plants (Baasanmunkh et al. 2022). Thus, it is important to study grasshopper's habitat and development, which negatively impact grassland. At the same time, there is an enormous shortage of taxonomists who can identify and describe species (Wheeler 2004). The loss of diversity coupled with the taxonomic impediment is one of the most challenging issues we biologists face today (Song 2010). A poor understanding of grasshopper fauna has impaired our understanding of grasshopper speciation and evolution. Therefore, further in-depth surveys of grasshoppers in Mongolia should be conducted and it is expected that the taxonomic uncertainty checked in this study can be solved through future studies.

Notably, some species are widely distributed in neighbouring countries, such as Russia, China and South Korea. The taxonomic keys of the superfamily Acridoidea in Mongolia were recently updated and it was reported that the superfamily includes three families, 49 genera and 127 species (Batkhuyag and Batnaran 2021). A difference of six species was observed between our checklist and the list updated by Batkhuyag and Batnaran (2021). These species included *Gomphoceruslicenti* Chang, Chorthippus (Ch.) karelini Uv., *Stenobothruskirgizorum* Ikonn., *Sphingonotushalocnemi* Uv., *Sphingonotusturcmenus* B.-Bien. and *Eclipophlepslevis* Mistsh. In addition, the records of specimens of four species were uncertain; these included Chorthippus (G.) buyanticus Batkhuyag et al., Chorthippus (G.) tseelicus Batlhuyag et al., *Sphingonotushalocnemi* Uv. and *Sphingonotusturcmenus* B.-Bien. In the present study, four species had uncertain distribution sources, namely *Gomphoceruslicenti* Chang, Chorthippus (Ch.) karelini Uv., *Stenobothruskirgizorum* Ikonn. and *Eclipophlepslevis* Mistsh and were excluded from our checklist. We are aware that this list is only a basis for further research and we hope that it will be further modified by the work of future scientists who devote their time and passion to researching new and interesting facts about Mongolian grasshoppers.

## Supplementary Material

30AE4B21-C0B8-5C22-8837-CA5002C47EC3 10.3897/BDJ.11.e96705.suppl1 Supplementary material 1 Species list of grasshoppers’ geographical natural distribution in six types of zones Data type table Brief description Captions: (*) = Endemic of Mongolia, (+) = geographical distribution natural zone, (★) = Indicator species of geographical natural zones, (-) = poor species. Abbreviation: 1-High mountain, 2-Taiga, 3-Forest steppe, 4-Steppe, 5-Desert steppe, 6-Desert natural zone. File: oo_796377.docx https://binary.pensoft.net/file/796377 Altanchimeg

75DDE256-9C81-5BAE-B146-CE87BD565189 10.3897/BDJ.11.e96705.suppl2 Supplementary material 2 Species registered in the European Red List of grasshopper Data type table File: oo_738113.docx https://binary.pensoft.net/file/738113 Enkhtsetseg

02F076DE-A563-53F0-8FFE-5BEF51BCCCA2 10.3897/BDJ.11.e96705.suppl3 Supplementary material 3 Shannon and Berger Parker’s index of natural zone Data type table File: oo_738114.docx https://binary.pensoft.net/file/738114 Enkhtsetseg

D6E53EEB-B3C7-5DB3-B993-95BBE397BA51 10.3897/BDJ.11.e96705.suppl4 Supplementary material 4 Shannon and Berger-Parker index of neighbouring boundary countries grasshopper distribution Data type table File: oo_738115.docx https://binary.pensoft.net/file/738115 Enkhtsetseg

## Figures and Tables

**Table 1.** Similarity matrix of grasshopper in six types of natural zones in Mongolia.

Natural zone	High Mountain (%)	Taiga (%)	Forest steppe (%)	Steppe (%)	Desert steppe (%)	Desert (%)
High Mountain	*	12.12	11.11	26.37	29.17	21.88
Taiga	*	*	46.58	28.26	20.62	12.31
Forest steppe	*	*	*	59.54	36.76	25.00
Steppe	*	*	*	*	64.52	40.65
Desert steppe	*	*	*	*	*	64.06
Desert	*	*	*	*	*	*

Cluster analysed amongst six different types of natural zone shown in %.

**Table 2.** Composition of grasshoppers (Acridoidea) in Mongolia.

No.	Subfamily	Tribe	Genus	Number of Species	Registered in the European red list
1	Acridinae MacLeay, 1821	Acridini MacLeay, 1821	*Acrida* Linnaeus, 1758	1	-
2	Gomphocerinae Fieber, 1853	Arcypterini Bolívar, 1914	*Arcyptera* Serville, 1838	4	2
6			*Andrea* Mistshenko, 1989	1	-
7		Chrysochraontini Brunner von Wattenwyl, 1893	*Chrysochraon* Fischer, 1853	1	-
8			*Euthystira* Fieber, 1852	1	1
9			*Mongolotettix* Rehn, 1928	3	-
12			*Podismopsis* Zubovski, 1900	2	-
14		Dociostaurini Mistshenko, 1974	*Eremippus* Uvarov, 1926	3	1
17			*Notostaurus* Bey-Bienko, 1933	1	1
18		Hypernephiini Mistshenko, 1973	*Eclipophleps* Tarbinsky, 1927	8	-
26		Gomphocerini Fieber, 1853	*Chorthippus* Fieber, 1852	16	8
42			*Aeropedellus* Hebard, 1935	4	1
46			*Pseudochorthippus* Defaut, 2012	2	
48			*Gomphocerippus* Roberts, 1941	1	1
49			*Gomphocerus* Thunberg, 1815	1	1
50			*Myrmeleotettix* Bolívar, 1914	2	-
52			*Stauroderus* Bolívar, 1897	1	1
53			*Schmidtiacris* Storozhenko, 2002	1	-
54			*Mesasippus* Tarbinsky, 1931	1	-
55			*Dasyhippus* Uvarov, 1930	1	-
56			*Dociostaurus* Fieber, 1853	2	1
58	Oedipodinae Walker, 1871	Stenobothrini Harz, 1975	*Omocestus* Bolívar, 1878	5	4
63			*Stenobothrus* Fischer, 1853	5	3
68			*Megaulacobothrus* Caudell, 1921	1	
69			*Leptopternis* Saussure, 1884	2	1
71		Bryodemini Bey-Bienko, 1930	*Bryodema* Fieber, 1853	6	-
77			*Compsorhipis* Saussure, 1889	3	-
80			*Bryodema* Yin, 1982	5	-
85			*Angaracris* Bey-Bienko, 1930	1	-
86		Epacromiini Brunner von Wattenwyl, 1893	*Aiolopus* Fieber, 1853	1	-
87			*Epacromius* Uvarov, 1942	2	1
89		Locustini Kirby, 1825	*Oedaleus* Fieber, 1853	3	-
92			*Locusta* Linnaeus, 1758	1	1
93			*Psophus* Fieber, 1853	1	1
94		Oedipodini Walker, 1871	*Celes* Saussure, 1884	1	-
95		Parapleurini Brunner von Wattenwyl, 1893	*Stethophyma* Fischer, 1853	1	-
96		Sphingonotini Johnston, 1956	*Sphingoderus* Bey-Bienko, 1950	1	1
97			*Sphingonotus* Fieber, 1852	11	3
108			*Helioscirtus Saussure, 1884*	1	
109	Melanoplinae Scudder, 1897	Podismini Jacobson, 1905	*Bohemanella* Ramme, 1951	1	-
110			*Podisma* Berthold, 1827	1	-
111			*Prumna* Motschulsky, 1859	1	-
112			*Ognevia* Ikonnikov, 1911	1	-
113			*Zubovskya* Dovnar-Zapolskij, 1932	2	-
115	Calliptaminae Jacobson, 1905	Calopteni Brunner von Wattenwyl, 1893	*Calliptamus* Serville, 1831	3	1
118	Egnatiinae Bey-Bienko & Mistshenko, 1951	Egnatiini Bey-Bienko & Mistshenko, 1951	*Egnatioides* Vosseler, 1902	1	
119	Dericorythinae Jacobson and Bianchi, 1905	Derocorythini Jacobson & Bianchi, 1905	*Dericorys* Serville, 1838	1	-
120	Thrinchinae Stål, 1876	Thrinchini Stål, 1876	*Beybienkia* Tsyplenkov, 1956	2	-
123			*Mongolotmethis* Bey-Bienko, 1948	3	-
125			*Rhinotmethis* Sjöstedt, 1933	2	-
127			*Asiotmethis* Uvarov, 1943	1	-
128		Haplotropiidini Sergeev, 1995	*Haplotropis* Saussure, 1888	1	-
Total	8	19	52	128	34

**Table 3.** Similarity matrix of grasshopper distribution in neighbouring countries (China, Russia and South Korea).

Name of Country	Mongolia (%)	South Korea (%)	Russia (%)	China (%)
Mongolia	*	26.85	76.56	68.37
South Korea	*	*	36.00	36.78
Russia	*	*	*	65.31
China	*	*	*	*

## References

[B8157210] Adelung NN (1906). Materials on the fauna and flora of the Russian empire.. Zoology.

[B8156536] Adelung N. (1910). Horae Societatis Entomologicae Rossicae, variis sermonibus in Rossia usitatis editae.

[B8082287] Altanchimeg D., Nonnaizab N. (2005). Study on the karyotype of *Bryodemaholdereri* (Acridoidea). Proceedings of Institute of Biology.

[B8082175] Altanchimeg D. (2011). Acridoidea of Mongolia.

[B8156517] Altanchimeg D., Nonnaizab N. (2013). Grasshoppers (Acridoidea) of Mongolian Plateau.

[B8082109] Altanchimeg D., Lin Chen, Nonnaizab N. (2013). Comparative study on the karyotype of two species of *Megaulacobothrus* Caud., 1921 (Acridoidea). Mongolian Journal of Biological Sciences.

[B8082118] Altanchimeg D., Uranbileg G., Unurzaya Kh., Zulbayr M., Dorjderem S. (2013). Grasshoppers (Acridoidea) of Khan khentii protected area.

[B8082100] Altanchimeg D., Chen L., Nonnaizab N. (2014). A new species of the genus *Aeropedellus* from the Hovsgol province of Mongolia (Orthoptera: Acrididae: Gomphocerinae). Transactions of the American Entomological Society.

[B8082056] Altanchimeg D., Enkhnasan D., Arigunsudar P. (2015). Insect biodiversity of Gurvan tes district, Omnogovi province. Proceedings of Institute of Biology.

[B8331474] Baasanmunkh S, Urgamal M, Oyuntsetseg B, Sukhorukov A. P, Tsegmed Z, Son D. C, Erst A, Oyundelger K, Kechaykin A. A, Norris J, Kosachev P, Ma J. S, Chang K. S, Choi H. J (2022). Flora of Mongolia: annotated checklist of native vascular plants. PhytoKeys.

[B8130232] Baccetti B. (1959). Notulae Orthopterologicae. X. Ricerche sugli Ortotteridel Gran Sasso d’Italia per il Centro di Entomologia Alpina. Redia.

[B8170057] Batkhuyag B (1995). Studying the biology and ecology of the main pest grasshoppers and pest management in Mongolia. Thesis of Ph.D.

[B8082091] Batkhuyag B., Batnaran Kh., Dorjderem S. (2014). A new species of *Mongolotmethis* from the Gobi Region of Mongolia (Orthoptera: Pamphagidae). Journal of Orthoptera Research.

[B8081973] Batkhuyag B., Bakey A., Batchuluun Ye., Chimeddorj B., Dagvasuren M., Davaadorj G., Gombobaatar S., Munkhchuluun B (2019). Sixth national report to the convention on biological diversity (2015-2018).

[B8081920] Batkhuyag B., Batnaran B. (2021). Key of the short-horned orthopteroid insects in Mongolia..

[B8082332] Batnaran B., Mendjargal B., Davaasuren J., Chogsomzhav L (1999). Conference of "Memories of the year" dedicated L. Chogsomjav.

[B8082254] Batnaran Kh. (2008). Studying the biology and ecology of some pest grasshoppers and pest control in central Mongolia.Thesis of Ph.D..

[B8082028] Batnaran Kh., Batkhuyag B., Otgonchimeg T., Dorjderem S., Turbat T., Gandulam R. (2016). A study on the karyotype of some pest grasshoppers in Mongolia.. Report of Science and Technology Foundation.

[B8331493] Batsaikhan N, Buuveibaatar B, Chimed B, Enkhtuya O, Galbrakh D, Ganbaatar O, Lkhagvasuren B, Nandintsetseg D, Berger J, Calabrese Justin M (2014). Conserving the world's finest grassland amidst ambitious national development. Conservation Biology.

[B8082156] Bazelet Corinna S., Samways Michael J. (2012). Grasshopper and butterfly local congruency in grassland remnants. Journal of Insect Conservation.

[B8082323] Benediktov A. A. (1999). To little-known taxa of *Chorthippusbiguttulus* group (Orthoptera, Acrididae, and Gomphocerinae). Vestnik Moskovskogo Universiteta Seriya.

[B8082019] Benediktov A. A. (2016). Variability of wings pattern of the locusts from the tribe *Bryodemini* Bey-Bienko (Orthoptera: Acrididae).

[B8130223] Bey-Bienko G. Ya (1926). Notes on some Orthoptera from Palaearctic Asia. Transactions Siberian Academic Agriculture Forest.

[B8082785] Bey-Bienko G. Ya (1930). A monograph of the genus *Bryodema* Fieb. (Orthoptera, Acrididae) and its nearest allies.. Annuaire du Musée Zoologique de l'Académie Impériale des Sciences de Sant-Pétersbourg.

[B8082767] Bey-Bienko G. Ya (1932). Notes on the genus *Compsorhipis* Sauss. (Orthoptera: Acrididae).. A Journal of Taxonomic Entomology.

[B8082758] Bey-Bienko G. Ya (1933). Orthoptera collected by Prof. V. Baranov in north western Mongolia.. Boletín de la Real Sociedad Española de Historia Natural.

[B8082722] Bey-Bienko G. Ya (1941). New and little known Orthoptera found in the USSR.. Zapiski Leningradskogo Selskokhozjastvennogo.

[B8082713] Bey-Bienko G. Ya (1948). Grasshoppers of the tribe Thrinchini (Orthoptera, Acrididae) collected by Russian investigators in Mongolia and limitrophic China. Entomologicheskoe Obozrenie.

[B8170086] Bey-Bienko G. Ya, Mistshenko L. L, Staff Translated and Edited by: IPST, Technical Editorial Consultant: Robert Lathan Randell Macdonald College, McGill University, Canada. Research Associate, Academy of Natural Sciences of Philadelphia, Philadelphia, Pa. , U. S. A. (1951). Locusts and Grasshoppers of the U.S.S.R. and Adjacent Countries..

[B8086293] Bi Daoying (1986). Description of five new grasshoppers from China (Orthoptera: Acridoidea). Contributions from the Shanghai Institute of Entomology.

[B8082953] Bolívar I. (1898). Contributions à l'étude des Acridiens espèces de la Faune indo et austro-malaisienne du Museo Civico di Storia Naturale di Genova. Annali del Museo Civico di Storia Naturale di Genova.

[B8082926] Bolívar I. (1901). In Zichy. Zoologische Ergebnisse der Dritten Asiatischen Forschungsreise des Grafen Eugen Zichy.

[B8082628] Cejchan A., Maran J. (1966). Orthoptera aus der Mongolischen Volksrepublik. Zugleich ergebnisse der Mongolisch-Deutschen biologischen expedition seit 1962. Nr. 11. Mitteilungen aus dem Zoologischen Museum in Berlin.

[B8082305] Childebaev M. K., Storozhenko S. Y (2001). An annotated list of brachycerous orthopterous insects (Orthoptera: Caelifera) occurring in Kazakhstan. Tethys Entomological Research.

[B8102937] Chogsomzhav L., Shurovenkov (1963). Fauna of grasshoppers (Orthoptera, Acrididae) of the Mongolian People's Republic. - 5th meeting.

[B8082610] Chogsomzhav L. (1968). The distribution of Mongolian grasshopper (Acrididae, Orthoptera).. Journal of University of Agriculture, Mongolia.

[B8082584] Chogsomzhav L. (1969). New record of orthopteroid insect.

[B8082575] Chogsomzhav L. (1969). Study of Orthoptera. Proceedings of Institute of Biology.

[B8082566] Chogsomzhav L. (1970). Orthoptera in the basin of the great lakes of the Mongolian People's Republic (Orthoptera).. Proceedings of Institute Biology.

[B8082557] Chogsomzhav L. (1971). Acridoidea and Tettigonioidea of Mongolian People's Republic.. Insects of Mongolia.

[B8091263] Chogsomzhav L. (1972). Acridoidea and Tettigonioidea of the Mongolian People’s Republic.. Insects of Mongolia.

[B8082504] Chogsomzhav L. (1974). A new species of the genus *Mongolotettix* Rehn (Orthoptera, Acrididae) from Mongolia.. Entomological Review.

[B8082513] Chogsomzhav L. (1974). Orthopteroid insects (Orthopteroidea) of western and southern Mongolia..

[B8082486] Chogsomzhav L. (1975). Orthopteroidea collected by the entomological group of the Soviet-Mongolian complex biological expedition in the year 1971. Insects of Mongolia.

[B8082469] Chogsomzhav L. (1977). Orthopteroidea of the Gobi Desert.. Insects of Mongolia.

[B8082389] Chogsomzhav L. (1989). Composition and distribution of fauna of the Orthopteroidea in the Mongolian People's Republic. Insects of Mongolia.

[B8130300] Chopard L. (1949). Note sur les Orthoptéroïdes du Sahara marocain. Bulletin of the Society of Natural Sciences of Morocco.

[B8081875] Cigliano MM, Braun H, Eades DC, Otte D (2022). Orthoptera species file. Version 5.0/5.0.. http://Orthoptera.SpeciesFile.org.

[B8087205] Creutzer C. (1799). Entomologische Versuche.

[B8087286] De Geer (1773). Mémoires pour servir à l'histoire des insectes, Pierre Hesselberg, Stockholm.

[B8081911] Dey Lara-Sophie, Seidel Matthias, Lchagvasuren Davaa, Husemann Martin (2021). From the steppe to the desert: Survey of band-winged grasshoppers from Mongolia (Orthoptera: Acrididae: Oedipodinae) based on material from 50 Years of expeditions. Erforschung Biologischer Ressourcen der Mongolei/ Exploration into the Biological Resources of Mongolia.

[B8082047] Fang Jingyun, Bai Yongfei, Wu Jianguo (2015). Towards a better understanding of landscape patterns and ecosystem processes of the Mongolian Plateau. Landscape Ecology.

[B8082147] Fartmann Thomas, Krämer Benjamin, Stelzner Friederike, Poniatowski Dominik (2012). Orthoptera as ecological indicators for succession in steppe grassland. Ecological Indicators.

[B8087060] Fieber F. X. (1853). Synopsis der Europaischen Orthopteren mit besonderer Rücksicht der Böhmischen Arten.. Lotus (Prag).

[B8087078] Fischer Leopold Heinrich (1849). Beitraege zur Insekten-fauna Freiburgs, Orthoptera. Jahresbericht, Mannheimer Verein für Naturkunde.

[B8083009] Fischer von Waldheim G. (1836). Orthoptera duo e montibus Catunicis descripta et icone illustrata.. Bulletin de la Société Impériale des Naturalistes de Moscou.

[B8082991] Fischer von Waldheim G. (1846). Entomographia Imperii Rossici. IV. Orthoptera Imperii Rossici.. Nouveaux Mémoires de la Société Impériale des Naturalistes de Moscou.

[B8086743] Furukawa H. (1929). Some alpine orthopterans from Mt. Ogahana, with description of a new subspecies.. Kontyu.

[B8082700] Furukawa H., Shiraki T, Omachi F, Shiraki T (1950). Orthoptera.

[B8086043] Ganbold Ya. (2009). Study of medicinal properties from raw materials of wide distributed Mongolian Acridoidea grasshopper and to produced preparation technology “Acritract”.

[B8082011] Gandulam R. (2016). Species composition of grasshopper (Acrididae) in the Khentii province.

[B8082296] Garai A. (2001). Orthopteroid insects of the Adrienne Garai and Péter Gyulai expedition to Mongolia in 1997. Esperiana.

[B8086007] Garai A. (2010). Contribution to the knowledge of the Iranian Orthopteroid insects I. Esperiana.

[B8087234] Gmelin J. F. (1789). Caroli a Linné, Systema naturae per regna tria naturae, secundum classes, ordines, genera, species; cum characteribus, differentiis, synonymis, locis. Editio decimo tertia, aucta, reformata. G.E. Beer, Lipsiae [= Leipzig].

[B8082083] Gombobaatar S., Myagmarsuren S., Conaboy N., Мunkhjargal M. (2014). Convention on biological diversity: The 5th national report of Mongolia. https://www.cbd.int/doc/world/mn/mn-nr-05-en.pdf.

[B8082380] Gorochov A. V., Mistshenko L. L, Podgornaya L. I (1989). Materials on the fauna and ecology of Orthoptera of the Transaltai Gobi. Nasekomye Mongolii [Insects of Mongolia].

[B8082548] Günther K. K. (1971). Blattoidea-Orthopteroidea-Ausbeute 1964, Teil II (Tetrigidae und Acrididae). Ergebnisse der Mongolisch-Deutschen Biologischen Expeditionen seit 1962, Nr. 55.. Mitteilungen aus dem Zoologischen Museum in Berlin.

[B8130980] Gupta V. K. (1983). The locust and grasshopper agricultural manual 1982. Oriental Insects.

[B8331508] Herbert Hurka, Nikolai Friesen, Karl-Georg Bernhardt, Barbara Neuffer Sergej, Smirnov Alexander Shmakov, Frank Blattner (2019). The Eurasian steppe belt: Status quo, origin and evolutionary history. Turczaninowia.

[B8082478] Hewitt George B. (1977). Review of forage losses caused by rangeland grasshoppers.

[B8081985] Hodjat Seyed Hossein, Tork Mehdi, Seiedy Marjan, Defaut Bernard (2018). A taxonomic review of recorded species of Caelifera (Orthoptera) in Iran. Matériaux orthoptériques et entomocénotiques.

[B8086355] Huang Chunmei (1981). Orthoptera: Acrididae, Catantopinae, Pyrgomorphinae, Oedipodinae. In: Insects of Xizang. Science Press, Beijing.

[B8082891] Ikonnikov N. (1911). Zur Kenntnis der Acridiodeen Sibiriens.. Annuaire du Musée Zoologique de l'Académie Impériale des Sciences de Saint-Pétersbourg.

[B8085682] IUCN (2022). The IUCN red list of threatened species. Version 2022-September.. https://www.iucnredlist.org.Accessedon[Feb,2022]..

[B8104076] Ivanova I. V. (1967). On the fauna of Orthoptera of the southern part of the Krasnojarsk Region, Central Siberia.. Entomologicheskoe Obozrenie.

[B8086928] Jacobson G. G., Bianchi. V. L. (1905). Orthopteroid and pseudoneuropteroid insects of the Russian empire and adjacent countries.

[B8082362] Jin Xingbao (1994). Genus *Haplotropis* Saussure. In Xia, K.-L. & et al. Acridoidea: Pamphagidae, Chrotogonidae, Pyrgomorphidae.. Fauna Sinica, Insecta.

[B8086901] Karny H. H. (1908). Orthoptera. A Dictyoptera, Tettigonioidea, Acridoidea. Wissenschaftliche Ergebnisse der Expedition Filchner nach China und Tibet 1903-1905..

[B8318085] Kietzka, Lecoq Gabriella J, Samways Michel, J Michael (2021). Ecological and human diet value of locusts in a changing world. Agronomy.

[B8087069] Kittary M. (1849). Orthoptères observés dans les steppes des Kirguises par MM. le Professeur P. Wagner et le Docteur Kittary, en 1846, déterminés et décrits. Bulletin de la Société Impériale des Naturalistes de Moscou.

[B8082917] Krauss H. A. (1901). Orthopteren vom Kuku-nor-Gebiet in Centralasien, gesammelt von Dr. J. Holderer im Jahre 1898.. Zoologischer Anzeiger.

[B8082165] Latchininsky Alexandre, Sword Gregory, Sergeev Michael, Cigliano Maria Marta, Lecoq Michel (2011). Locusts and Grasshoppers: Behavior, Ecology, and Biogeography. Psyche-A journal of Entomology.

[B8318113] Lecoq Michel, Zhang Long (2019). Encyclopedia of pest Orthoptera of the world.

[B8318122] Lecoq Michel, Cease Arianne (2022). What Have We Learned after Millennia of Locust Invasions?. Agronomy.

[B8086311] Lee Hyeung Sik, Lee Chang Eon. (1985). Taxonomic revision of the Catantopinae from Korea (Orthoptera: Acrididae) IV. Calliptamini and Eyprepocnemidini.. The Korean Journal of Entomology.

[B8086221] Lee H. S., Park. W. H. (1992). A new genus of the subfamily Oedipodinae (Orthoptera: Acrididae). Basic Science Research Institute of Hyosung Women's University.

[B8086320] Z Lian, Zheng Z (1984). New genera and new species of grasshoppers from Gansu, China.. Entomotaxonomia.

[B8086346] Li Hongchang. (1981). Studies on the fauna of the genus *Angaracris* B.-Bienko (Orthoptera: Acrididae). Acta Zootaxonomica Sinica.

[B8082270] Li Hongchang., Hao. S., Kang. L. (2007). Regional differentiation of the Acridoidea ecofaunas in different vegetational zones (subzones) of Inner Mongolia region. Acta Entomologica Sinica.

[B8082245] Lockwood Dale R, Lockwood Jeffrey A (2008). Grasshopper population ecology: catastrophe, criticality, and critique. Ecology and Society.

[B8082236] Marini Lorenzo, Fontana Paolo, Scotton Michele, Klimek Sebastian (2008). Vascular plant and Orthoptera diversity in relation to grassland management and landscape composition in the European Alps. Journal of Applied Ecology.

[B8086858] Matsumura S. (1911). Erster Beitrag zur Insekten-Fauna von Sachalin.. The Journal of the College of Agriculture, Tohoku Imperial University, Sapporo, Japan.

[B8082451] Miksic S. (1981). Mitteleuropäische und mediterrane Orthopteren in der Fauna des herzegowinischen Karstes.. Acta Entomological Jugoslavica.

[B8082731] Mistshenko L. L (1937). Revision of palaearctic species of the genus *Sphingonotus* Fieber (Orthoptera: Acrididae). Eos, Revista Española de Entomología.

[B8082682] Mistshenko L. L, Bey-Bienko G. Ya (1951). Keys to the fauna of the U.S.S.R. [1964 English translation no. 40]. Locusts and grasshoppers of the U.S.S.R. and adjacent countries.

[B8082673] Mistshenko L. L (1952). Locusts and grasshoppers, Catantopinae.. Fauna of the U.S.S.R..

[B8082601] Mistshenko L. L (1968). Orthopteroid insects (Orthopteroidea) collected by the entomological expedition of the zoological institute, USSR academy of sciences in the Mongolian People’s Republic in 1967.. Entomological Review.

[B8082521] Mistshenko L. L (1973). Grasshoppers of the genus *Eclipophleps* S. Tarb. (Orthoptera: Acrididae). Entomological Review.

[B8082416] Mistshenko L. L, Gorochov A. V. (1989). In Gorochov, Mistshenko & Podgornaya. Materials on the fauna and ecology of Orthoptera of the Transaltai Gobi. Nasekomye Mongolii [Insects of Mongolia].

[B8085889] Mohamed A, Kimura R (2014). Applying the moisture availability index (NTDI) over vegetated land in Central Asia: Mongolian steppe.. Journal of Water Resource and Protection.

[B8082184] Munkhbat J. (2010). Study of Orthoptera insect communities from Hustai National Park. https://biology.mn/?p=product&viewby=single&id=112#description.

[B8081964] Myagmar G., Dorzhiev Ts. Z., Gantigmaa Ch. (2019). The fauna of orthopteran insects of the Galba desert in the south eastern Mongolia.

[B8157254] Nonnaizab NA, Qi B, Li Y (1999). Insects of Inner Mongolia China.

[B8087226] Olivier G. A. (1791). Criquet, Acrydium. In: Olivier M, Encyclopédie méthodique. Histoire naturelle. Insectes.

[B8082655] Orishchenko. (1960). *Eclipophlepstarbinskii* is een rechtvleugelig insect uit de familie veldsprinkhanen (Acrididae).. De wetenschappelijke naam van deze soort is voor het eerst geldig gepubliceerd in 1960 door Orishchenko.

[B8081994] Otgonchimeg T. (2017). Study of grasshopper (Acrididae) species composition and distribution patterns of forest-steppe zone of Mongolia.

[B8082407] Podgornaya L. I., Gorochov A. V. (1989). In Gorochov, Mistshenko & Podgornaya. Materials on the fauna and ecology of Orthoptera of the Transaltai Gobi.. Nasekomye Mongolii [Insects of Mongolia].

[B8081946] Popova Kristina V., Molodtsov Vladimir V., Sergeev Michael G. (2020). Rare grasshoppers (Orthoptera, Acridoidea) of the Baraba and Kulunda steppes (South Siberia). Acta Biologica Sibirica.

[B8158148] Predtechenskii (1928). Notes of the Astrakhan plant protection station against pests.

[B8082864] Pylnov E. (1916). Contributions à la faune des Acridoidea et des Locustodea de la Mongolie boréale.. Russian Entomological Review.

[B8081900] Qian Hongge, Altanchimeg D., Naizab Non, Wang Shusen, Wen Suyaletu, Lin Chen (2021). The complete mitochondrial genome of *Eclipophlepscarinata* (Orthoptera: Acridoidea: Gomphoceridae). Mitochondrial DNA Part B.

[B8086671] Ramme W. (1939). Beiträge zur Kenntnis der palaearktischen Orthopteren fauna (Tettig. u. Acrid.) III. Mitteilungen aus dem Zoologischen Museum in Berlin.

[B8086595] Ramme W. (1952). Orthopteren der Sven Hedin-Expedition nach China 1927-30. Ergänzungen und Berichtigungen zur Bearbeitung durch Y. Sjöstedt 1933.. Archive for Zoology.

[B8086990] Redtenbacher J. (1889). Beitrag zur Orthopteren-Fauna von Turkmenien.. Wiener Entomologische Zeitschrift.

[B8081937] Rentsendorj Gandulam, Khodroi Batnaran (2020). Study review of the composition grasshoppers in Mongolia. Mongolian Journal of Agricultural Sciences.

[B8130263] Saussure Henri (1884). Prodromus oedipodiorum insectorum ex ordine orthopterorum.

[B8130271] Saussure Henri (1888). Additamenta ad prodromum Oedipodiorum.

[B8082345] Sergeev M. G. (1995). The general distribution of Orthoptera in the eastern parts of the Saharan-Gobian and Scythian subregions.. Acta Zoologica Cracoviensia.

[B8082219] Sergeev M. G., Jirong Murav’eva V. M., N.E. Hudiakova (2009). Diversity and distribution patterns of Orthoptera in the Altai Mountains.. Amurian Zoological Journal.

[B8081955] Sergeev M. G., Storozhenko S. Y, Benediktov A. A (2019). An annotated check-list of Orthoptera of Tuva and adjacent regions. Part 2. Suboder Caelifera. Tridactylidae, Tetrigidae, Acrididae: Melanoplinae, Calliptaminae, and Gomphocerinae (except Gomphocerini).. Far Eastern Entomologist.

[B8081928] Sergeev M. G., Storozhenko S. Y, Benediktov A. A (2020). An annotated check-list of Orthoptera of Tuva and adjacent regions. Part 3. Suboder Caelifera (Acrididae: Gomphocerinae: Gomphocerini, Locustinae).. Far Eastern Entomologist.

[B8081891] Sergeev M. G. (2021). Distribution patterns of grasshoppers and their kin over the Eurasian steppes. Insects.

[B8331528] Song Hojun (2010). Grasshopper Systematics: Past, Present and Future. Journal of Orthoptera Research.

[B8087034] Stål C. (1861). Orthoptera species novas descripsit.. Kongliga Svenska Fregatten Eugenies Resa Omkring Jorden under befäl af C.A. Virgin åren 1851-1853 (Zoologi).

[B8082637] Steinmann H. (1964). Ergebnisse der zoologischen Forschungen von Dr. Z. Kaszab in der Mongolei 20. Tetrigidae und Acrididae (Orthoptera).. Folia Entomologica Hungarica.

[B8082619] Steinmann H. (1967). Tetrigidae and Acridiidae. Ergebnisse der zoologischen Forschungen von Dr. Z. Kaszab in der Mongolei 99. (Orthoptera).. Reichenbachia.

[B8082592] Steinmann H. (1968). Tetrigidae und Acridiidae. Ergebnisse der zoologischen Forschungen von Dr. Z. Kaszab in der Mongolei (Orthoptera).. Reichenbachia.

[B8082539] Steinmann H. (1971). Tetrigidae und Acrididae. Ergebnisse der zoologischen Forschungen von Dr. Z. Kaszab in der Mongolei (Orthoptera).. Faunistische Abhandlungen. Staatliches Museum für Tierkunde in Dresden.

[B8083052] Stoll C. (1813). Représentation exactement colorée d'après nature des spectres ou phasmes, des mantes, des sauterelles, des grillons, des criquets et des blattes, qui se trouvent dans les quatre parties du monde, l'Europe, l'Asie, l'Afrique et l'Amerique.

[B8082434] Storozhenko S. Y (1985). New representatives of the genus *Stenobothrus* (Orthoptera, Acrididae) from the Sovjet Far East.. Zoologicheskij Zhurnal.

[B8082425] Storozhenko S. Y (1986). Revision of the genus *Zubovskya* Dov.-Zap. (Orthoptera, Acrididae).. Zoological Institute (Leningrad), Academy of Sciences SSSR.

[B8082262] Storozhenko S. Y, Paik J. - Ch. (2007). Orthoptera of Korea.

[B8082039] Storozhenko Sergey Y, Kim Tae Woo, Jeon Mi (2015). Monograph of Korean Orthoptera.

[B8087256] Sulzer J. H. (1776). Abgekuerzte Geschichte der Insecten nach dem Linnei’schen System.

[B8082847] Tarbinsky S. P. (1925). Zur Kenntnis der Gattung Chorthippus Fieb. (Orthoptera: Acridinae). Konowia.

[B8156527] Tarbinsky S. P. (1927). On some new and little-known Orthoptera from Palaearctic Asia.. Annals and Magazine of Natural History.

[B8082776] Tarbinsky S. P. (1931). A revision of the Palaearctic species of the genera *Gomphocerus* Thunb. and Dasyhippus Uvar. (Acrididae). Bulletin of the Leningrad Institute for Controlling Farm and Forest Pests..

[B8082210] Tishechkin D. Yu, Bukhvalova M. A (2009). New data on and calling signal of Gomphocerinae grasshopper (Orthoptera: Acrididae) from South Siberia and Russian Far East. Russian Entomological Journal.

[B8082664] Tsyplenkov EP (1956). A new genus of the tribe Thrinchini (Orthoptera, Acrididae) from western China. Entomologicheskoe Obozrenie, Moscow.

[B8082065] Uchida Kei, Ushimaru Atushi (2014). Biodiversity declines due to abandonment and intensification of agricultural lands: patterns and mechanisms. Ecological Monographs.

[B8082002] Ünal (2016). Pamphagidae (Orthoptera: Acridoidea) from the Palaearctic Region: taxonomy, classification, keys to genera and a review of the tribe Nocarodeini I. Bolívar.. Zootaxa.

[B8156544] Uvarov BP (1910). Contributions sur la faune des Orthoptères de la province de l’Oural. Horae Societatis entomologicae Rossicae.

[B8082873] Uvarov B. P. (1914). Contributions to the fauna of orthoptera in the province of Transbaikalia.. Directory of the Zoological Museum of the Imperial Academy of Sciences in Saint Petersburg..

[B8082740] Uvarov B. P. (1934). Studies in the Orthoptera of Turkey, Iraq and Syria.. Revista Española de Entomología.

[B8318190] Werner Franz (1905). Ergebnisse einer zoologischen Forschungsreise nach Ägypten und dem ägyptischen Sudan. I. Die Orthopterenfauna Ägyptens mit besonderer Burücksichtigung der Eremiaphilen. Sitzungsberichte der kaiserlichen Akademie der Wissenschaften, Mathematisch -naturwissenschaftliche Klasse, Wien.

[B8331519] Wheeler Quentin D (2004). Taxonomic triage and the poverty of phylogeny. Philosophical Transactions of the Royal Society of London. Series B: Biological Sciences.

[B8082201] Willemse Luc P. M. (2009). In Çiplak, K.-G. Heller & F.M.H. Willemse. Review of the genus *Eupholidoptera* (Orthoptera, Tettigoniidae): different genitalia, uniform song.. Zootaxa..

[B8086778] Wnukowskij W. (1926). Zur Fauna der Orthopteren und Dermapteren des Bezirks Kamenj (südwestliches Sibirien, früheres Gouvernement Tomsk). Mitteilungen der Münchner Entomologischen Gesellschaft.

[B8130952] Worden Robert L., Savada Andrea Matles (1991). Mongolia: A country study.

[B8085698] Yembuu Batchuluun editor (2021). The physical geography of Mongolia.

[B8082460] Yin X. - C., Chou. (1979). Two new genera and three new species of Acrididae from Shaanxi province.. Entomotaxonomia.

[B8086102] Yin X. - C., Wang Wenqiang (2005). A new species of Compsorhipis Saussure (Orthoptera, Acrididae, Oedipodinae), with a key to the known species from China and adjacent areas. http://www.biodiversitylibrary.org/item/113865#page/31/mode/1up.

[B8157270] Zhang Dao-Chuan, Wang Wen-Qiang, Yin Xiang-Chu (2006). A new species of *Bryodema* (Orthoptera: Acridoidea) from China, with a key to the described species. Entomological News.

[B8318094] Zhang Long, Lecoq Long, Latchininsky Michel, Hunter Alexandre, David (2019). Locust and grasshopper management. Annual Review of Entomology.

[B8086212] Zheng Z., Ren. G. (1993). Four new species of grasshoppers from northern west of China (Orthoptera: Acridoidea).. Journal of Hubei University (Natural Science)..

[B8086194] Zheng Z., He. D. (1994). Two new species of grasshoppers from Ningxia (Orthoptera: Acridoidea).. Journal of Hubei University (Natural Science)..

[B8086168] Zheng Z., Han. Yali (1998). Two new species of grasshoppers (Orthoptera: Acridoidea) from Nei Mongol.. Entomotaxonomia.

[B8082137] Zheng Z., Zeng Hui-Huai, Zhang Hong-Li, Tao S., Su. S. (2012). A survey of grasshoppers from Helan mountain national reserve in Inner Mongolia (Orthoptera).. Journal of Shaanxi Normal University (Natural Science Edition).

[B8082935] Zubovski N (1900). Beitrag zur Kenntniss der sibirischen Acridiodeen. Trudy Russkago Entomologicheskago Obshchestva [Horae Societatis Entomologicae Rossicae]..

