# Supplementary material for: An annotated checklist of grasshoppers (Orthoptera, Acridoidea) from Mongolia
Source: Biodivers Data J. 2023 Mar 13;11:e96705. doi: 10.3897/BDJ.11.e96705 (PMC10848637; doi:10.3897/BDJ.11.e96705)
Supplement: Supplementary material 1 — Species list of grasshoppers’ geographical natural distribution in six types of zones [file bdj-11-e96705-s001.docx]

Supplementary Table 1. Species list of grasshoppers’ geographical natural distribution in six types of zones. Captions: (*)= Endemic of Mongolia; (+)=geographical distribution natural zone; (★)= Indicator species of geographical natural zones; (-)= poor species. Abbreviation: 1-High Mountain; 2-Taiga; 3-Forest steppe; 4-Steppe; 5-Desert steppe; 6-Desert natural zone.

Species list of grasshoppers’ geographical natural distribution in six types of zones

| № | Name of species | Geographical distribution natural zone | | | | | |
| --- | --- | --- | --- | --- | --- | --- | --- |
|  |  | 1 | 2 | 3 | 4 | 5 | 6 |
| 1 | *Asiotmethis similis* B.-Bien. | - | - | + | - | ★ | - |
| 2 | *Beybienkia lithophila* Gor. - Mishch. | - | - | - | - | ★ | - |
| 3 | *Beybienkia songorica* Tsy. | - | - | - | - | - | + |
| 4 | *Mongolotmethis gobiensis* B.-Bien. * | - | - | - | + | ★ | - |
| 5 | *Mongolotmethis kozlovi* B.-Bien. * | - | - | - | + | ★ | - |
| 6 | *Mongolotmethis michidi* Batnaran et al. | - | - | - | + | ★ | - |
| 7 | *Rhinotmethis beybienkoi* Chogsomzhav | - | - | - | - | ★ | ★ |
| 8 | *Rhinotmethis hummeli* Sjost. * | - | - | - | - | ★ | ★ |
| 9 | *Haplotropis brunneriana* Sauss. | - | - | + | + | - | - |
| 10 | *Dericorys annulata* (Fieb.) | - | - | - | - | ★ | - |
| 11 | *Bohemanella frigida* (Boh.) | - | ★ | ★ | - | - | - |
| 12 | *Podisma pedestris* (L.) | - | ★ | ★ | - | - | - |
| 13 | *Prumna primnoa* Mots. | - | - | ★ | + | - | - |
| 14 | *Zubovskya mongolica* Storozhenko | - | - | + | - | - | - |
| 15 | *Zubovskya koeppeni* (Zub.) | - | - | + | - | - | - |
| 16 | *Calliptamus abbreviatus* Ikonn. | + | - | + | + | + | + |
| 17 | *Calliptamus barbarus cephalotes* F.-W. | - | - | - | + | ★ | ★ |
| 18 | *Calliptamus italicus* L. | - | - | - | + | - | - |
| 19 | *Acrida kozlovi* Mistsh. | - | - | - | - | ★ | ★ |
| 20 | *Chrysochroan dispar* (Germ.) | + | + | + | + | + | + |
| 21 | *Eutystira brachyptera* (Ocsk.) | + | + | + | + | + | + |
| 22 | *Mongolotettix japonicus* (Bol.) | - | + | + | + | + | - |
| 23 | *Mongolotettix mistshenkoi* Chogsomzhav | - | - | - | + | + | - |
| 24 | *Mongolotettix vittatus* Uv. | - | - | - | + | - | - |
| 25 | *Podismopsis altaica* Zub. * | + | - | - | + | + | - |
| 26 | *Podismopsis ussuriensis* Ikonn. | - | - | + | - | - | - |
| 27 | *Eclipophleps bogdanovi* Tarb. * | ★ | - | - | + | + | - |
| 28 | *Eclipophleps carinata* Mistsh. * | ★ | - | - | - | - | + |
| 29 | *Eclipophleps confinis* Mistsh. * | ★ | - | - | + | + | - |
| 30 | *Eclipophleps glacialis* B.-Bien. * | ★ | - | - | + | + | - |
| 31 | *Eclipophleps kerzhneri* Mistsh. * | ★ | - | - | + | + | + |
| 32 | *Eclipophleps lucida* Mistsh. * | ★ | - | - | + | + | + |
| 33 | *Eclipophleps similis* Mistsh. * | ★ | - | - | + | + | - |
| 34 | *Eclipophleps tarbinskii* Oristsh. * | ★ | - | - | + | + | - |
| 35 | *Arcyptera albogeniculata* Ikonn. | - | - | ★ | - | - | - |
| 36 | *Arcyptera fusca* (Pall.) | - | - | + | - | - | - |
| 37 | *Arcyptera meridionalis* Ikonn. | - | - | ★ | ★ | ★ | - |
| 38 | *Arcyptera microptera* (F.-W.) | - | - | ★ | ★ | ★ | - |
| 39 | *Eremippus mistshenkoi* Steb. | - | - | - | + | - | - |
| 40 | *Eremippus mongolicus* Rme. | - | - | - | + | + | + |
| *…continued the next page* | | | | | | | |
| Continued. | | | | | | | |
| № | Name of species | Geographical distribution natural zone | | | | | |
|  |  | 1 | 2 | 3 | 4 | 5 | 6 |
| 41 | *Eremippus simplex* Mistsh. | - | - | - | + | + | + |
| 42 | *Dociostaurus brevicollis* (Ev.) | - | - | + | + | + | + |
| 43 | *Dociostaurus (Kazakia) tarbinskyi* B.-Bien. | - | - | - | - | - | + |
| 44 | *Notostaurus albicornis* (Ev.) | - | - | - | + | + | + |
| 45 | *Aeropedellus baliolus* Mistsh. | - | - | - | + | - | - |
| 46 | *Aeropedellus chogsomjavi* Altanchimeg et al. | - | - | - | + | - | - |
| 47 | *Aeropedellus reuteri* Mir. | - | - | - | + | + | - |
| 48 | *Aeropedellus variegatus* (F.-W.) | - | - | + | + | - | - |
| 49 | *Chorthippus (A.) fallax* (Zub.) | - | + | + | + | + | - |
| 50 | *Chorthippus (A.) hammarstroemi* (Mir.) | - | + | + | + | + | + |
| 51 | *Chorthippus (A.) intermedius* B.-Bien. | - | + | ★ | ★ | - | - |
| 52 | *Chorthippus (Ch.) albomarginatus* (De Geer) | - | + | + | + | + | + |
| 53 | *Chorthippus (Ch.) dichrous* (Ev.) | + | - | + | + | + | + |
| 54 | *Chorthippus (Ch.) dorsatus* Zett. | - | - | + | + | + | - |
| 55 | *Chorthippus (Ch.) ilkazi* Uv. | - | - | - | + | + | - |
| 56 | *Chorthippus (Ch.) turanicus* Tarb. | - | - | - | + | - | - |
| 57 | *Chorthippus (G.) apricarius* (L.) | - | - | + | + | - | - |
| 58 | *Chorthippus biguttulus* (L.) | - | + | + | + | + | - |
| 59 | *Chorthippus (G.) brunneus* (Thunb.) | - | + | + | + | + | - |
| 60 | *Chorthippus (G.) dubius* Zub. | - | - | + | + | + | - |
| 61 | *Chorthippus (G) maritimus* Mistsh. | + | - | - | - | + | - |
| 62 | *Chorthippus (G.) mollis* (Charp.) | - | - | - | - | + | + |
| 63 | *Chorthippus (G.) vagans* (Ev.) | - | - | + | - | - | - |
| 64 | *Chorthippus (M.) chinensis* Tarb. | - | - | + | - | - | - |
| 65 | *Chorthippus (Ch.) caliginosus* Mistsh. | - | - | + | - | - | - |
| 66 | *Dasyhippus barbipes* (F.-W.) | - | - | - | + | + | + |
| 67 | *Pseudochorthippus* *montanus* (Charp.) | - | + | + | + | + | - |
| 68 | *Pseudochorthippus parallelus* (Zett.) | - | - | - | + | - | - |
| 69 | *Gomphocerus rufus* (L.) | - | - | + | + | - | - |
| 70 | *Gomphocerus sibiricus* (L.) | - | - | ★ | + | - | - |
| 71 | *Mesasippus kozhevnikovi robustus* (Tarb.) | - | - | - | - | + | + |
| 72 | *Myrmeleotettix palpalis* (Zub.) | - | - | + | + | + | + |
| 73 | *Myrmeleotettix zaitzevi* Mistsh. | - | - | - | - | + | - |
| 74 | *Omocestus haemorrhoidalis* (Charp.) | - | - | + | + | - | - |
| 75 | *Omocestus petraeus* Bris. | - | - | - | + | - | - |
| 76 | *Omocestus rufipes* (Zett.) | - | - | - | - | - | - |
| 77 | *Omocestus tzendsureni* Gunth. | - | - | + | + | + | - |
| 78 | *Omocestus viridulus* (L.) | - | - | + | + | + | + |
| 79 | *Schmidtiacris schmidti* (Ikonn.) | - | - | + | - | - | - |
| 80 | *Stauroderus scalaris* (F.-W.) | - | - | + | + | - | - |
| 81 | *Stenobothrus carbonarius* (Ev.) | - | - | + | - | - | - |
| 82 | *Stenobothrus eurasius* Zub. | - | - | ★ | ★ | - | - |
| 83 | *Stenobothrus fischeri* Ev. | - | - | - | - | ★ | ★ |
| 84 | *Stenobothrus lineatus* Panz. | - | - | + | + | - | - |
| 85 | *Stenobothrus newskii* Zub. * | - | ★ | ★ | - | - | - |
| 86 | *Stethophyma grossus* L. | - | + | + | + | - | - |
| 87 | *Aiolopus thalassinus* (Fabr.) | - | - | - | ★ | - | - |
| *…continued the next page* | | | | | | | |
| Continued. | | | | | | | |
| № | Name of species | Geographical distribution natural zone | | | | | |
|  |  | 1 | 2 | 3 | 4 | 5 | 6 |
| 88 | *Epacromius pulverulenthus* (F.-W.) | - | - | + | - | - | - |
| 89 | *Epacromius tergestinus* (M-M.) | - | - | ★ | + | + | + |
| 90 | *Locusta migratoria* (L.) | - | - | - | - | + | + |
| 91 | *Oedaleus asiaticus* B.-Bien. | - | - | - | ★ | + | - |
| 92 | *Oedaleus decorus* (Germ.) | - | - | - | + | - | + |
| 93 | *Oedaleus infernalis* Sauss. | - | - | - | + | + | - |
| 94 | *Ognevia longipennis* (Shir.) | - | ★ | ★ | - | - | - |
| 95 | *Psophus stridulus* (L.) | - | - | ★ | + | - | - |
| 96 | *Andrea gorochovi* Mistsh. | - | - | - | - | + | - |
| 97 | *Angaracris barabensis* (Pall.) | - | - | + | ★ | + | + |
| 98 | *Bryodema gebleri* (F.-W.) * | - | - | - | + | + | + |
| 99 | *Bryodema heptapotanicum* B.-Bien. | - | - | - | - | + | - |
| 100 | *Bryodema kozlovi* B.-Bien. | - | - | - | - | + | - |
| 101 | *Bryodema luctuosum* (Stoll.) | - | - | - | ★ | + | + |
| 102 | *Bryodema miramae* B.-Bien. | - | - | - | - | + | - |
| 103 | *Bryodema nigripennis* Mistsh. et al. | - | - | - | - | + | - |
| 104 | *Bryodema (B.) holdereri* Kr. | - | - | ★ | ★ | + | + |
| 105 | *Bryodema (B.) tuberculate* (Stoll) | - | + | ★ | ★ | + | - |
| 106 | *Bryodema (M.) orientalis* B.-Bien. * | - | - | - | + | + | + |
| 107 | *Bryodema (M.) semenovi* Ikonn. | + | - | - | - | - | - |
| 108 | *Bryodema (M.) zaisanicum fallax* B.-Bien. | - | - | + | - | - | - |
| 109 | *Celes skalozubovi* Adel. | - | - | ★ | + | + | + |
| 110 | *Compsorhipis bryodemoides* B.-Bien. * | - | - | - | - | ★ | ★ |
| 111 | *Compsorhipis davidiana* (Sauss.) | - | - | - | - | ★ | ★ |
| 112 | *Compsorhipis orientalis* Chogs. | - | - | - | + | + | - |
| 113 | *Leptopternis gracilis* (Ev.) | - | - | - | - | ★ | ★ |
| 114 | *Leptopternis iliensis* Uv. | - | - | - | - | - | + |
| 115 | *Sphingoderus carinatus* (Sauss.) | - | - | - | - | ★ | ★ |
| 116 | *Sphingonotus beybienkoi* Mistsh. | - | - | - | + | ★ | ★ |
| 117 | *Sphingonotus coerulipes* Uv. | - | - | - | - | ★ | - |
| 118 | *Sphingonotus elegans* Mistsh. | - | - | - | - | ★ | ★ |
| 119 | *Sphingonotus gobicus* Chogs.* | - | - | - | - | ★ | ★ |
| 120 | *Sphingonotus mongolicus* Sauss. | - | + | + | + | - | - |
| 121 | *Sphingonotus nebulosus* (F.-W.) | - | - | - | - | ★ | - |
| 122 | *Sphingonotus obscuratus latissimus* Uv. | - | - | - | - | ★ | + |
| 123 | *Sphingonotus rubescens* (Walker) | + | - | - | - | ★ | - |
| 124 | *Sphingonotus salinus* (Pall.) | - | - | - | - | ★ | + |
| 125 | *Sphingonotus tzaidamicus* Mistsh. | - | - | - | - | ★ | + |
| 126 | *Sphingonotus halophilus* B.-Bien. | - | - | - | - | + | + |
| 127 | *Egnatioides desertus* Uv. | - | - | - | - | - | + |
| 128 | *Helioscirtus moseri* Sauss. | - | - | - | - | - | + |
|  | SubTotal | 16 | 17 | 56 | 75 | 80 | 48 |
|  | Total | 292 | | | | | |
