# Supplementary material for: An annotated checklist of grasshoppers (Orthoptera, Acridoidea) from Mongolia
Source: Biodivers Data J. 2023 Mar 13;11:e96705. doi: 10.3897/BDJ.11.e96705 (PMC10848637; doi:10.3897/BDJ.11.e96705)
Supplement: Supplementary material 2 — Species registered in the European Red List of grasshopper [file bdj-11-e96705-s002.docx]

Supplementary Table 2. Species registered in the European Red List of Grasshopper.

Three Near Threatened (NT) species; two Endangered (EN) species; twenty-eight Least Concern (LC) species were registered.

| 1 | *Aeropedellus variegatus* (F.-W.) | EN |
| --- | --- | --- |
| 2 | *Arcyptera fusca* (Pall.) | LC |
| 3 | *Arcyptera microptera* (F.-W.) | LC |
| 4 | *Calliptamus italicus* L. | LC |
| 5 | *Chorthippus (Ch.) albomarginatus* (De Geer) | LC |
| 6 | *Chorthippus (Ch.) dichrous* (Ev.) | LC |
| 7 | *Chorthippus (Ch.) dorsatus* Zett. | LC |
| 8 | *Chorthippus (G.) apricarius* (L.) | LC |
| 9 | *Chorthippus (G.) brunneus* (Thunb.) | LC |
| 10 | *Chorthippus (G.) dubius* Zub | DD |
| 11 | *Chorthippus (G.) mollis* (Charp.) | LC |
| 12 | *Chorthippus (G.) vagans* (Ev.) | LC |
| 13 | *Chorthippus biguttulus* (L.) | LC |
| 14 | *Dociostaurus brevicollis* (Ev.) | LC |
| 15 | *Epacromius tergestinus* (M-M.) | LC |
| 16 | *Eremippus simplex* Mistsh. | NT |
| 17 | *Gomphocerippus rufus* (L.) | LC |
| 18 | *Gomphocerus sibiricus* (L.) | LC |
| 19 | *Leptopternis gracilis* (Ev.) | LC |
| 20 | *Locusta migratoria* (L.) | LC |
| 21 | *Notostaurus albicornis* (Ev.) | LC |
| 22 | *Omocestus haemorrhoidalis* (Charp.) | LC |
| 23 | *Omocestus petraeus* Bris. | LC |
| 24 | *Omocestus rufipes* (Zett.) | LC |
| 25 | *Omocestus viridulus* (L.) | LC |
| 26 | *Psophus stridulus* (L.) | LC |
| 27 | *Sphingoderus carinatus* (Sauss.) | NT |
| 28 | *Sphingonotus coerulipes* Uv. | LC |
| 29 | *Sphingonotus rubescens* (Walker) | LC |
| 30 | *Sphingonotus salinus* (Pall.) | EN |
| 31 | *Stauroderus scalaris* (F.-W.) | LC |
| 32 | *Stenobothrus carbonarius* (Ev.) | NT |
| 33 | *Stenobothrus eurasius* Zub. | LC |
| 34 | *Stenobothrus lineatus* Panz | LC |
|  | *Total* | 34 |
