# Supplementary material for: An annotated checklist of grasshoppers (Orthoptera, Acridoidea) from Mongolia
Source: Biodivers Data J. 2023 Mar 13;11:e96705. doi: 10.3897/BDJ.11.e96705 (PMC10848637; doi:10.3897/BDJ.11.e96705)
Supplement: Supplementary material 3 — Shannon and Berger Parker’s index of natural zone [file bdj-11-e96705-s003.docx]

Table S3. Shannon index of Natural zone

| Index | High Mountain | Taiga | Forest steppe | Steppe | Desert steppe | Desert |
| --- | --- | --- | --- | --- | --- | --- |
| Shannon H' Log Base 10. | 1.204 | 1.23 | 1.74 | 1.869 | 1.892 | 1.653 |
| Shannon Hmax Log Base 10. | 1.204 | 1.23 | 1.74 | 1.869 | 1.892 | 1.653 |
| Shannon J' | 1 | 1 | 1 | 1 | 1 | 1 |

Table S4. Berger Parker’s index of Natural zone.

| Index | High Mountain | Taiga | Forest steppe | Steppe | Desert steppe | Desert |
| --- | --- | --- | --- | --- | --- | --- |
| Berger-Parker Dominance (d) | 0.063 | 0.059 | 0.018 | 0.014 | 0.013 | 0.022 |
| Berger-Parker Dominance (1/d) | 16 | 17 | 53 | 74 | 78 | 44 |
| Berger-Parker Dominance (d%) | 6.25 | 5.882 | 1.818 | 1.351 | 1.282 | 2.222 |
