# Supplementary material for: An annotated checklist of grasshoppers (Orthoptera, Acridoidea) from Mongolia
Source: Biodivers Data J. 2023 Mar 13;11:e96705. doi: 10.3897/BDJ.11.e96705 (PMC10848637; doi:10.3897/BDJ.11.e96705)
Supplement: Supplementary material 4 — Shannon and Berger-Parker index of neighbouring boundary countries grasshopper distribution [file bdj-11-e96705-s004.docx]

Table S5. Shannon index of neighboring boundary countries grasshopper distribution.

| Index | Mongolia | China | South Korea | Russia |
| --- | --- | --- | --- | --- |
| Shannon H' Log Base 10. | 2.083 | 1.813 | 1.362 | 1.886 |
| Shannon Hmax Log Base 10. | 2.083 | 1.813 | 1.362 | 1.886 |
| Shannon J' | 1 | 1 | 1 | 1 |

Table S6. Berger-Parker index of neighboring boundary countries grasshopper distribution.

| Index | Mongolia | China | South Korea | Russia |
| --- | --- | --- | --- | --- |
| Berger-Parker Dominance (d) | 0.008 | 0.015 | 0.043 | 0.013 |
| Berger-Parker Dominance (1/d) | 121 | 65 | 23 | 77 |
| Berger-Parker Dominance (d%) | 0.826 | 1.538 | 4.348 | 1.299 |
